# Circulating levels of cytokines and risk of inflammatory bowel disease: evidence from genetic data

**DOI:** 10.3389/fimmu.2023.1310086

**Published:** 2023-12-11

**Authors:** Bin Liu, Yu Qian, Yanan Li, Xiangting Shen, Ding Ye, Yingying Mao, Xiaohui Sun

**Affiliations:** ^1^ Department of Epidemiology, Zhejiang Chinese Medical University School of Public Health, Hangzhou, China; ^2^ Diseases & Population (DaP) Geninfo Lab, School of Life Sciences, Westlake University, Hangzhou, Zhejiang, China

**Keywords:** interleukin-17, monokine induced by interferon-gamma, Mendelian randomization, inflammatory bowel disease, single nucleotide polymorphism

## Abstract

**Background:**

Prior epidemiological studies have established a correlation between inflammatory cytokines and inflammatory bowel disease (IBD). However, the nature of this relationship remains uncertain. Mendelian randomization (MR) study has the advantages of avoiding confounding and reverse causality compared with traditional observational research.

**Objective:**

We aimed to evaluate whether genetically determined circulating levels of cytokines are associated with the risk of IBD by using the MR approach.

**Materials and methods:**

We selected genetic variants associated with circulating levels of 28 cytokines at the genome-wide significance level from a genome-wide association study (GWAS) including 8,293 individuals. Summary-level data for IBD (including Crohn’s disease and ulcerative colitis) were obtained from the International Inflammatory Bowel Disease Genetics Consortium and UK Biobank. We performed the primary analysis using the inverse-variance weighted method, as well as sensitivity analyses to test the stability of our results. We subsequently replicated the results of IBD in the UK Biobank dataset. A reverse MR analysis was also conducted to evaluate the possibility of reverse causation.

**Results:**

Genetically predicted elevated levels of interleukin-17 (IL-17) and monokine induced by interferon-gamma (MIG) were associated with an increased risk of IBD[odds ratio (OR): 1.52, 95% confidence interval (CI):1.10-2.08, *P* =0.010 for IL-17 and OR: 1.58, 95% CI: 1.24-2.00, *P* = 1.60×10^-4^ for MIG]. Moreover, we observed suggestive associations between β-NGF and MIP-1β with the risk of Crohn’s disease (OR: 0.71, 95% CI: 0.52-0.98, *P* = 0.039) and ulcerative colitis (OR: 1.08, 95% CI: 1.01-1.15, *P*= 0.019). In the reverse MR study, there was no evidence of causal effects of IBD and these cytokines.

**Conclusion:**

Our study suggests the potential causal associations of IL-17 and MIG with IBD. Further studies are needed to determine whether IL-17 and MIG or their downstream effectors could be useful in the management of IBD.

## Introduction

Inflammatory bowel disease (IBD), comprising Crohn’s disease (CD) and ulcerative colitis (UC), is a chronic immune-mediated disease and characterized by episodes of recurrent abdominal pain, diarrhea, and rectal bleeding (1, 2). The occurrence of IBD in Western countries is progressively increasing at a stable rate, exhibiting an average yearly change of 2.86%(3). It is predicted that by 2030, the estimated prevalence of IBD will reach 981 per 100,000(3). Patients with IBD may have malnutrition, weight loss, and an increased risk of colorectal cancer, posing a formidable challenge to public health among aging populations (4).

The pathogenesis of IBD remains unclear, though both genetic and environmental factors (e.g., smoking, unhealthy diet patterns, and major depression) may contribute to its development (5, 6). Recently, increasing evidence indicated that inflammation, particularly cytokines, is intricately included in the development of IBD (7, 8). For example, previous epidemiological studies have reported that circulating levels of cytokines, such as interleukin (IL)-17, IL-18, IL-2 receptor, alpha subunit (IL-2rα), and monokine induced by interferon-gamma (MIG), were elevated in patients with IBD compared to control groups (9–13). However, observational studies are vulnerable to various sources of bias, including residual confounding and reverse causation(14). Recently, a genome-wide association study (GWAS) revealed that numerous signals associated with the risk of IBD are situated in proximity to genes implicated in immune response (i.e., *AHR*, *IL23R* and *PTGS2*)(15), but whether they are casual roles for IBD were still not fully elucidated. Thus, the potential causal relationships between specific cytokines and IBD need to be further explored.

Mendelian randomization (MR) is currently being extensively utilized to evaluate the potential causal relationship between risk factors and outcomes, utilizing genetic variants as instrumental variables (IVs) (16). Given that genetic variants remain unaffected by diseases and that alleles are randomly assigned to offspring, MR analysis is able to minimize the confounding and avoid reverse causation bias (17). Currently, two MR studies have identified the relationships between IL-18, IL-1ra and IBD (18, 19). However, some other important inflammatory markers have not been fully investigated. In this study, we systematically assessed potential causal relationships between circulating levels of 28 cytokines and the risk of IBD through two-sample MR analyses.

## Materials and methods

### Study design

**Figure 1:**
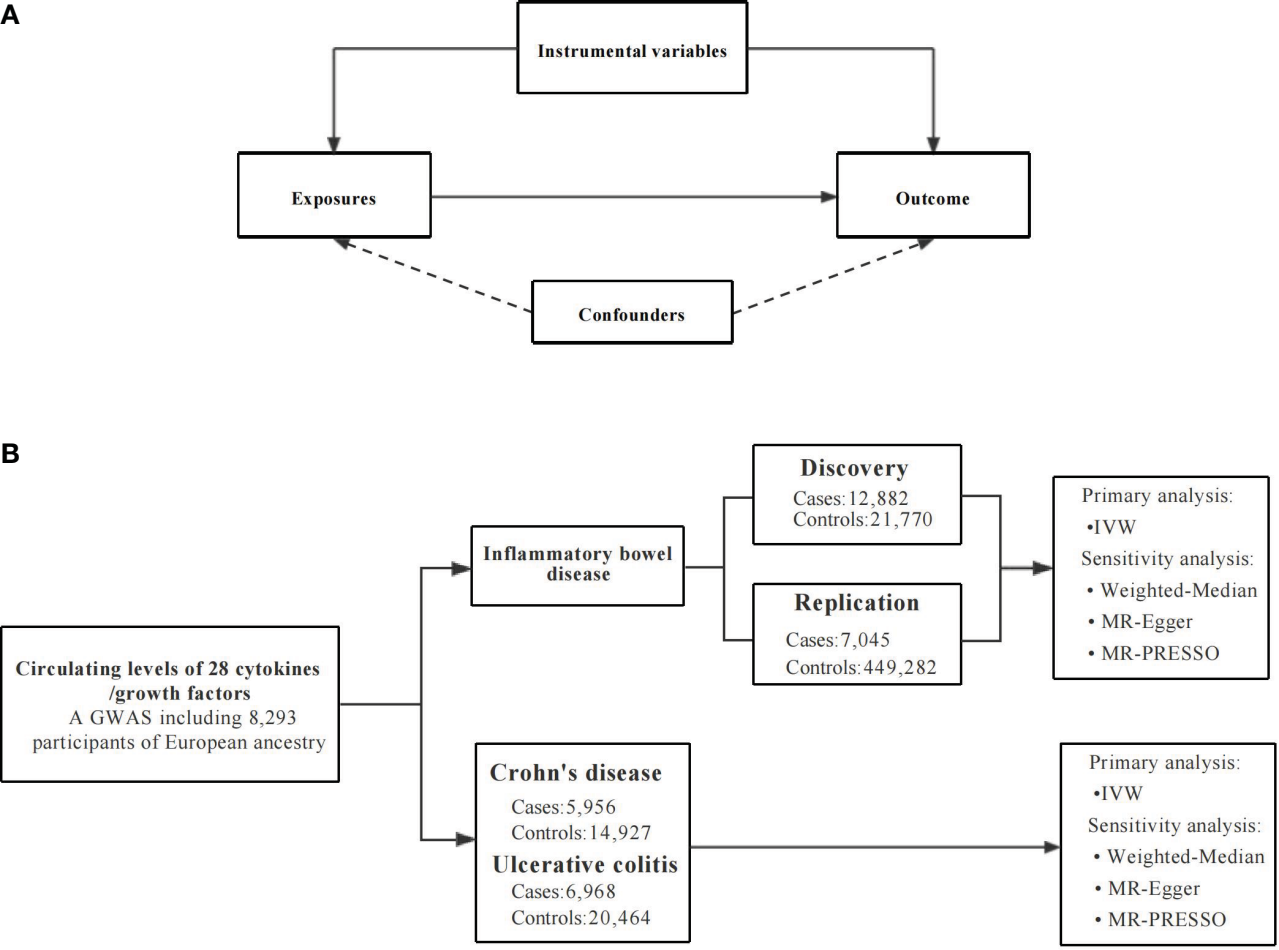
An overall design of the present study. GWAS, Genome-wide association study; MR, Mendelian randomization; MR-PRESSO, MR pleiotropy residual sum and outlier test; SNP, single nucleotide polymorphism. **(A)** The Mendelian randomization model and the three key assumptions. **(B)** The study flow diagram and data sources used in this study.

MR analysis demands to satisfy three assumptions (20). First, the genetic variants used as instrumental variables (IVs) are strongly related to circulating levels of cytokines (20). Secondly, the independent variables neither directly impact the outcome, nor do they affect the outcome through pathways other than the exposure itself (20). Third, the IVs should not be affected by any confounders (20). To satisfy these conditions, we set a series of strict standards for IV selection and employed several alternative MR methods. The overall design of the current study is depicted in **Figure 1**. Due to all GWAS summary statistics are publicly available, no additional ethical approval or informed consent from human participants was needed.

### Selection and description of cytokines

The summary data and IVs for cytokines were obtained from a GWAS which studied the genetic basis for 41 cytokines and growth factors involved 8,293 participants of European descent from The Cardiovascular Risk in Young Finns Study(21). This GWAS analysis was adjusted for age, sex, and the first ten genetic principal components and their effect sizes are in SD-scaled units (21).

For the IV selection, single nucleotide polymorphisms (SNPs) were chosen at the genome-wide significance (*P* < 5×10^-8^) and minor allele frequency (MAF) > 0.01 for their effect with circulating levels of cytokines. We pruned SNPs using a linkage disequilibrium (LD) threshold of r^2^ < 0.01 and a distance of 1,000kb, retaining the SNPs with the lowest *P*-values as IVs. In order to avoid the potential pleiotropy, we excluded the SNPs associated with more than one cytokine. We also manually searched each SNP in the GWAS Catalog (http://www.ebi.ac.uk/gwas, accessed on November 21th, 2023) and removed those associated with other phenotypes. After exclusion of these SNPs and 2 SNPs that were not found in the IBD (CD, UC) datasets, we used the remaining 122 SNPs as IVs for 28 cytokines in the subsequent MR analyses. The details of GWAS and the included SNPs are displayed in **Supplementary Table 1** and **Supplementary Table 2**.

### Selection and description of IBD

As shown in **Supplementary Table 1**, the summary data for IBD was acquired from a recent GWAS conducted by the International Inflammatory Bowel Disease Genetics Consortium (IIBDGC) (15). The IIBDGC of European cohort was consisted of BEL1, BEL2, CEDARS, CHOP, GERMAN, NIDDK, and WTCCC study. The diagnosis of IBD in this GWAS aligns with the clinically accepted criteria, involving radiological, endoscopic, and histopathological evaluations (15). The genetic associations were derived through the utilization of logistic regression with adjustment of age, sex, and genetic principal components. In our study, we only used summary statistics of European ancestry including IBD (12,882 cases, 21,770 controls), CD (5,956 cases, 14,927 controls) and UC (6,968 cases, 20,464 controls)(15). Furthermore, to confirm the reliability of our findings, replication was conducted using additional summary data of IBD sourced from the UK Biobank (UKB) including a total of 7,045 IBD cases and 449,282 controls. The patients with IBD in the UKB, composed of CD and UC, were defined according to death-register, self-reported, hospital admission or primary care record (22). However, this GWAS did not provide the summary data for CD and UC.

For the selection of IVs for IBD, CD and UC, we used the same criteria (*P* < 5×10^-8^, r^2^<0.01, distance = 1000 kb). In addition, we conducted a search within the GWAS Catalog (http://www.ebi.ac.uk/gwas, accessed on November 21th, 2023) for these IVs (**Supplementary Table 3**). After excluding the pleiotropic SNPs, 40 SNPs associated with IBD, 32 SNPs associated with CD and 31 SNPs associated with UC as IVs were retained for the reverse MR analysis (**Supplementary Table 4**).

### Statistical analysis

To assess the strength of the IVs, we calculated F-statistics using the following equation: F= β^2^/se^2^(23). In addition, the proportions of variances in 28 cytokines were estimated by R^2^ with the equation: R^2^ = 2 × MAF × (1−MAF) × β^2^ (24). The term “β” represents the effect size estimates of cytokine SNPs, “se” denotes the standard error associated with the SNPs, and “MAF” indicates the minimum allele frequency of the SNPs we utilized(24). We further utilized an online web tool (https://sb452.shinyapps.io/power/) to estimate the statistical power for the association of each cytokine with outcomes (25).

The inverse-variance-weighted (IVW) method was used as the primary analysis to evaluate the causal effect estimates. This method uses the effect size and standard error estimates of exposure and outcome and computes the individual MR estimates with the Wald ratio and Delta method (26). As sensitivity analyses, we additionally performed alternative MR analyses, encompassing the weighted-median method, MR-Egger regression, and the MR pleiotropy residual sum and outlier (MR-PRESSO) test. The weighted-median method can provide a valid estimate when assumed at least 50% of the variants involved in IVs are valid instruments(27). In addition, the MR-Egger regression was employed to detect potential directional pleiotropy, assessed by the P-value for the intercept (28). If the *P*-value < 0.05, it means that the IVs have potential directional pleiotropy (28). We also used the MR-PRESSO test to detect and obtain the corrected results after excluding possible outliers (29).

A *P*-value < 5.95×10^−4^ (0.05/28/3) was considered statistically significant evidence of a causal association, utilizing Bonferroni correction for 28 cytokines. A *P*-value ranging from 5.95×10^−4^ to 0.05 was considered as suggestive evidence (30). All analyses were performed in R software (v3.6.4) using “TwoSampleMR”, “Mendelian Randomization” and “MRPRESSO” (29, 31).

## Results

**Figure 2:**
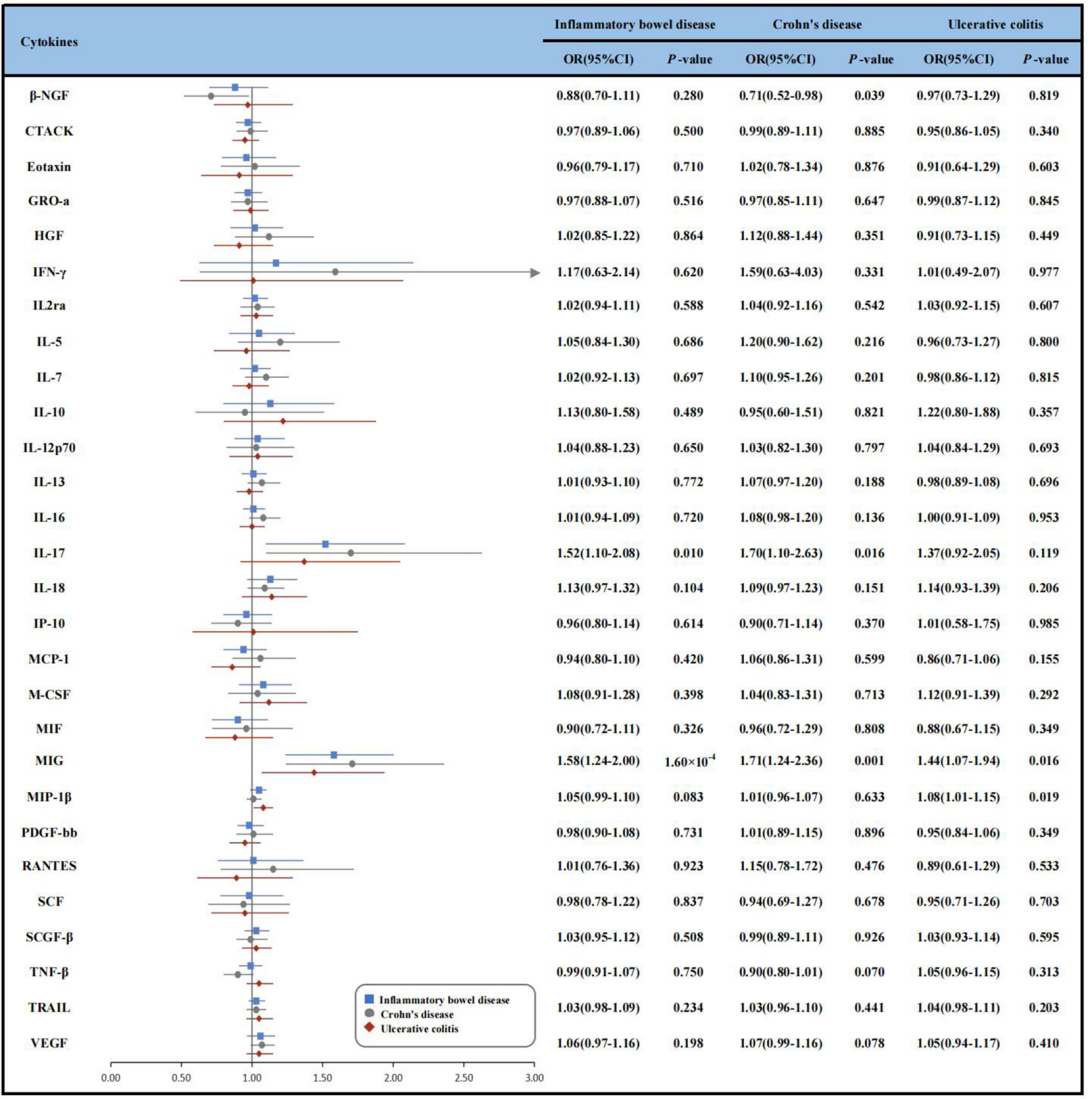
Forest plot of Mendelian randomization analyses for the associations between circulating cytokines and risk of inflammatory bowel disease, Crohn’s disease and ulcerative colitis. β-NGF, beta nerve growth factor; CD, Crohn’s disease; CI, confidence interval; CTACK, cutaneous T-cell attracting (CCL27); GRO-a, growth regulated oncogene-α (CXCL1); HGF, hepatocyte growth factor; IBD, inflammatory bowel disease; IFN-γ, Interferon-gamma; IL-2rα, interleukin-2 receptor, alpha subunit; IL-12p70, interleukin- 12p70; IL-13, interleukin-13; IL-16, interleukin-16; IL-17, interleukin-17; IL-18, interleukin-18;IP-10, Interferon gamma-induced protein 10 (CXCL10); MCP-1, monocyte chemotactic protein-1 (CCL2); M-CSF, macrophage colony-stimulating factor; MIF, macrophage migration inhibitory factor; MIG, monokine induced by interferon-gamma; MIP-1b, macrophage inflammatory protein-1β; PDGF-bb, platelet derived growth factor BB; RANTES, regulated on activation, normal T Cell expressed and secreted (CCL5); SCF, stem cell factor; SCGF-β, stem cell growth factor beta; SDF-1α, stromal cell-derived factor-1 alpha; SNP, single nucleotide polymorphism; TNF-β, tumor necrosis factor-beta; TRAIL, TNF-related apoptosis inducing ligand; UC, ulcerative colitis; VEGF, vascular endothelial growth factor; IVW, inverse-variance-weighted.

The F-statistics for the cytokines ranged from 22 to 789, meeting the threshold of >10 (**Supplementary Table 2**). Of the instruments analyzed, the most robust was for IL-7 with a median F-statistic of 169.84, while the least influential was for regulated on activation, normal t cell expressed and secreted with a median F-statistic of 29.99. The R^2^ values for the cytokines ranged from 0.002 (interferon-gamma) to 0.427 (macrophage inflammatory protein-1β, MIP-1β) (**Supplementary Table 2**). The IVs used for cytokines and the results of cytokines with IBD, CD, UC are presented in **Supplementary Table 5** and **Figure 2**.

### IBD

In the discovery analysis, we found significant associations between genetically determined higher levels of MIG with an increased risk of IBD. Since only one SNP was available as IV for MIG, we just conducted the IVW method and found that genetically determined higher levels of MIG were associated with a 58% higher risk of IBD, respectively [odds ratio (OR): 1.58, 95% confidence interval (CI):1.24-2.00, *P* = 1.60×10^-4^]. We also observed suggestive associations of IL-17 (OR: 1.52, 95% CI:1.10-2.08, *P* = 0.010) with the risk of IBD. Given the limited number of IVs, sensitivity analyses for the IL-17 were not available (**Supplementary Figure 1** and **Supplementary Table 6**). In addition, we replicated the associations of cytokines in the UKB dataset. Though the associations between IL-17 and MIG with IBD were not statistically significant, the directions of the effects were consistent (OR: 1.08, 95% CI: 0.78-1.49, *P* = 0.643 for IL-17 and OR: 1.13, 95% CI: 0.89-1.43, *P* = 0.316 for MIG) (**Supplementary Table 6**).

### CD

We identified 3 cytokines (β-NGF, IL-17, MIG) were suggestively associated with CD risk, and IL-17, MIG were also associated with IBD using IVW method (**Supplementary Figure 1**). Specifically, genetically determined higher levels of circulating IL-17 (OR: 1.70, 95% CI: 1.10-2.63, *P* = 0.016) and MIG (OR: 1.71, 95% CI: 1.24-2.36, *P*= 0.001) were suggestively associated with CD risk. However, genetically determined higher levels of beta nerve growth factor (β-NGF) were suggestive associated with 29% lower risk of CD (OR: 0.71, 95% CI: 0.52-0.98, *P* = 0.039). Other relationships between 25 cytokine and CD were listed in **Supplementary Table 7**.

### UC

As for UC, we observed that genetically determined elevated circulating levels of MIP-1β and MIG were suggestively associated with an increased risk of UC using IVW method (OR: 1.44, 95% CI: 1.07-1.94, *P* = 0.016 for MIG and OR: 1.08, 95% CI: 1.01-1.15, *P* = 0.019 for MIP-1β) (**Supplementary Table 8** and **Supplementary Figure 1**). The MR-PRESSO test also yielded similar results (OR: 1.08, 95% CI: 1.01-1.15, *P* = 0.024 for MIP-1β). MR-Egger regression analysis did not suggest directional pleiotropy for MIP-1β (intercept *P*-value = 0.626). However, the result of weighted-median method was not significance (OR: 1.03, 95% CI: 0.95-1.13, *P* = 0.463).

### Reverse MR analysis

Reverse MR analyses were further conducted to investigate the impacts of IBD, CD and UC on the identified significant cytokines. There was no evidence of causal effects of IBD on IL-17 (OR: 0.98, 95% CI: 0.94-1.02, *P* = 0.374) and MIG (OR: 1.01, 95% CI: 0.94-1.08, *P* = 0.864) using IVW method. In sensitivity analyses, the weighted median method, MR-PRESSO yielded similar estimates and there is no evidence of directional pleiotropy in MR-Egger regression (**Supplementary Table 9**).

For CD, we did not found reverse associations for these three cytokines (OR: 1.06, 95% CI: 1.00-1.13, *P*= 0.131 for β-NGF, OR: 1.00, 95% CI: 0.97-1.04, *P*= 0.820 for IL-17 and OR: 1.03, 95% CI: 0.97-1.10, *P*= 0.334 for MIG) (**Supplementary Table 10**). Furthermore, we did not observe statistically significant associations of UC with MIG (OR: 0.98, 95% CI: 0.93-1.03, *P*= 0.386) and MIP-1β (OR: 0.96, 95% CI: 0.92-1.00, *P*= 0.062) by IVW method (**Supplementary Table 11**). The results of the other sensitivity analyses are showed in **Supplementary Table 9** to **Supplementary Table 11**.

## Discussion

In our study, we used MR methods to systematically screen the causal associations of 28 cytokines with IBD (including CD and UC). We identified MIG was significantly associated with IBD risk, and IL-17 was suggestively associated. Additionally, the findings for the two IBD subtypes showed that circulating MIG was also suggestively linked with a higher risk of CD and UC. Furthermore, we observed potential links between elevated levels of circulating β-NGF, and IL-17 with the altered risk of CD. Moreover, we noticed a suggestive association between MIP-1β and UC. The reverse MR did not support their reverse associations.

MIG, a chemoattractant for Th1 cell which stimulate the expression of interferon-γ and tumor necrosis factor-α, was found mediating inflammation and tissue injury (32, 33). A population-based study consisting of 72 UC patients and 140 healthy controls found that the MIG was upregulated expression in patients with UC (*P*< 0.05), and this finding was further replicated in a twin-sibling study (34). Another study also reported a higher expression of MIG in 11 patients with UC (35). Consistent with previous research, our analysis demonstrated that genetically predetermined elevated levels of MIG was associated with an increased risk of developing IBD, CD and UC (11, 12). Though the association between MIG and IBD was not significant in UKB, the effect was similar. The recruitment of mononuclear cells and granulocytes by MIG may be the possible interpretation for the associations between MIG and IBD (11, 12). Moreover, MIG may infiltrate the CXCR3 axis to reach inflammatory sites in IBD, potentially contributing to the pathogenesis of the disease (11, 12). Reverse MR results did not support the association of IBD and CD with serum levels of MIG. Further studies are required to decipher the biological mechanism.

IL-17, the first member of IL-17 family of cytokines to be discovered and is characteristic feature of Th17 cells (36). For example, in Fujino’ s study, IL-17 expression in the mucosa and serum was found to be increased in IBD patients (37). The Tc17 cells have been identified as the primary source of IL17 in inflamed tissue and circulating Tc17 were found to be elevated in 122 IBD patients compared to 47 healthy controls (38, 39). In addition, animal experiments have demonstrated that the successful blockade of IL-17 can effectively alleviate intestinal inflammation in various mouse models of IBD (40). It has been documented that IL-17 has the ability to regulate RORγt, which undergoes various post-translational modifications, such as ubiquitination and phosphorylation, to initiate the inflammation associated with IBD. (41). Furthermore, cytokines belonging to the IL-17 family may contribute to the promotion of inflammation in barrier organs or inhibit intestinal repair (42). In addition, our reverse MR findings did not indicate that patients with IBD exhibit elevated serum levels of IL-17. Due to the limitations of observational studies such as the relatively small sample size and reverse causality, the MR findings in the present study have provided more reliable evidence for causal inference.

We also noticed suggestive evidence of associations between circulating β-NGF and the risk of CD, MIP-1β and the risk of UC. β-NGF, a neurotrophic factor involved in regulating neuronal survival and differentiation, might play an important role in inflammation (43). However, the evidence of the association between serum β-NGF levels and CD was limited. Mola et al. found the NGF mRNA levels increased 2.4 folds in CD patients compared with controls (44). However, our study found that a higher circulating level of β-NGF was associated with a lower risk of CD. MIP-1β,a type of MIP-1 protein that belongs to the chemokine family, is important in controlling inflammatory response (45). For instance, a case-control study including 26 patients with active UC (13 in the treatment group and 13 in the control group) showed that serum levels of MIP-1β in treated UC patients were significantly lower compared to those without treatment(46). This phenomenon could potentially arise from the substantial infiltration of monocytes into the epithelium, coupled with elevated levels of proinflammatory cytokines, including MIP-1β, in the context of active UC (47). However, due to the limited sample size especially for the cases, the associations between these cytokines and the risk of CD,UC needs a validation by further large-scale studies.

The current study has several strengths. First, the F-statistic of IVs satisfied the threshold of >10, which suggested that our results were less likely to suffer from weak instrument bias. Furthermore, although previous studies have assessed the relationship between some circulating cytokines and IBD risk, the cytokines they focused on were limited (only IL1-ra and IL-18) (18, 19). To the best of our knowledge, this is the first study to systematically investigate the associations between 28 cytokines and the risk of IBD (including CD and UC) and identify several potential causal cytokines. Moreover, we replicated our main results in another dataset, which broaden the samples and make our results more reliable.

Several limitations required to be noted when interpret our findings. Our findings might not be applied to other ethnicities due to the data used in our study were European ancestry. However, it minimized bias from population stratification. Second, while we employed summary data from the UKB for replication purposes, it is difficult for us to know the proportion of the overlapped sample in these two datasets. Further studies are warrened to replicate our findings in the independent datasets. Besides, we could not assess the potential nonlinear effects of cytokines on the risk IBD by using the MR method. Moreover, since we used a limited number of IVs for some cytokines, the power of them may be not enough to detect statistically significant associations. Finally, we did not obtain reliable genetic instruments for several important cytokines for IBD, such as IL-1β, IL-6 and TNF-α due to the stringent criterion. Thus, it is possible that we overlook the potential causality between these cytokines and the risk of IBD.

In conclusion, our MR study lends support to the potential causal associations of IL-17 and MIG with risk of IBD. Further studies are warranted in the future to explore the underlying biological mechanisms and confirm their potential as therapeutic targets.

## Data availability statement

The datasets presented in this study can be found in online repositories. The names of the repository/repositories and accession number(s) can be found in the article/**Supplementary Material**.

## Ethics statement

The summary-level data of our MR study was approved by their Institutional Review Board (IRB) or an equivalent committee and written informed consent was obtained from all participants.

## Author contributions

BL: Conceptualization, Data curation, Investigation, Methodology, Writing – original draft, Writing – review & editing. YQ: Conceptualization, Data curation, Formal Analysis, Methodology, Writing – original draft. YL: Data curation, Formal Analysis, Writing – original draft. XSh: Formal Analysis, Methodology, Writing – original draft. DY: Conceptualization, Data curation, Methodology, Project administration, Writing – original draft, Writing – review & editing. YM: Conceptualization, Investigation, Methodology, Resources, Supervision, Writing – original draft, Writing – review & editing. XSu: Conceptualization, Data curation, Formal Analysis, Funding acquisition, Investigation, Methodology, Resources, Writing – original draft, Writing – review & editing.
