# Supplementary material for: Circulating levels of cytokines and risk of inflammatory bowel disease: evidence from genetic data
Source: Front Immunol. 2023 Dec 11;14:1310086. doi: 10.3389/fimmu.2023.1310086 (PMC10750389; doi:10.3389/fimmu.2023.1310086)
Supplement: Supplementary file 1 [file DataSheet_1.docx]

Supplementary Material for

**Circulating levels of cytokines and risk of inflammatory bowel disease: evidence from genetic data**

**Supplementary Table 1** Details of the genome-wide association studies and datasets used in this study.

**Supplementary Table 2** Details of the numbers of genetic instruments, variance explained by the selected instruments and F-statistic for each cytokine and growth factor.

Abbreviations: No., number; SNP, single nucleotide polymorphism.

**Supplementary Table 3** Details of the genetic variants with potential pleiotropy among instrumental variables used for inflammatory bowel disease, Crohn's Disease, and ulcerative colitis.

Abbreviations: CD, Crohn's Disease; IBD, inflammatory Bowel Disease; SNP, single nucleotide polymorphism; UC, ulcerative colitis.

^*^ From the GWAS Catalog (http://www.ebi.ac.uk/gwas, last accessed on November 23th, 2023).

**Supplementary Table 4** Characteristics of the genetic variants associated with the inflammatory bowel disease, Crohn's Disease and ulcerative colitis.

Abbreviations: Chr, chromosome; CD, Crohn's Disease; IBD, inflammatory bowel disease; SE, standard error; SNP, single nucleotide polymorphism; UC, ulcerative colitis.

**Supplementary Table 5** Characteristics of instrumental variables used for circulating levels of 28 cytokines in this study.

**Supplementary Table 6** Effect estimates of the associations between circulating levels of 28 cytokines and risk of inflammatory bowel disease.

Abbreviations: CI, confidence interval; MR, Mendelian randomization; MR-PRESSO test, MR Pleiotropy RESidual Sum and Outlier test; OR, odds ratio; SNP, single nucleotide polymorphism. **P*-value of the intercept from MR-Egger regression analysis.

**Supplementary Table 7** Effect estimates of the associations between circulating levels of 28 cytokines and risk of Crohn's disease.

Abbreviations: CI, confidence interval; MR, Mendelian randomization; MR-PRESSO test, MR Pleiotropy RESidual Sum and Outlier test; OR, odds ratio; SNP, single nucleotide polymorphism. **P*-value of the intercept from MR-Egger regression analysis.

**Supplementary Table 8** Effect estimates of the associations between circulating levels of 28 cytokines and risk of ulcerative colitis.

Abbreviations: CI, confidence interval; MR, Mendelian randomization; MR-PRESSO test, MR Pleiotropy RESidual Sum and Outlier test; OR, odds ratio; SNP, single nucleotide polymorphism. **P*-value of the intercept from MR-Egger regression analysis.

**Supplementary Table 9** Effect estimates of the associations between inflammatory bowel disease and circulating levels of IL-17 and MIG.

Abbreviations: CI, confidence interval; IL-17, interleukin-17; MIG, monokine induced by interferon-gamma; MR-PRESSO test, MR Pleiotropy RESidual Sum and Outlier test; No., number of; OR, odds ratio; SNP, single nucleotide polymorphism; SNP, single nucleotide polymorphism. **P*-value of the intercept from MR-Egger regression analysis.

**Supplementary Table 10** Effect estimates of the associations of Crohn's disease with risk of circulating levels of β-NGF, IL-17 and MIG.

Abbreviations: β-NGF, beta nerve growth factor; CI, confidence interval; IL-17, interleukin-17; MIG, monokine induced by interferon-gamma; MR-PRESSO test, MR Pleiotropy RESidual Sum and Outlier test; No., number of; OR, odds ratio; SNP, single nucleotide polymorphism; SNP, single nucleotide polymorphism. **P*-value of the intercept from MR-Egger regression analysis.

**Supplementary Table 11** Effect estimates of the associations of ulcerative colitis with risk of circulating levels of MIG and MIP-1β.

Abbreviations: CI, confidence interval; MIG, monokine induced by interferon-gamma; MIP-1β, macrophage inflammatory protein-1β; MR-PRESSO test, MR Pleiotropy RESidual Sum and Outlier test; No., number of; OR, odds ratio; SNP, single nucleotide polymorphism; SNP, single nucleotide polymorphism. **P*-value of the intercept from MR-Egger regression analysis.

| **Supplementary Table 1** Details of the genome-wide association studies and datasets used in this study. | | | | | |
| --- | --- | --- | --- | --- | --- |
| Exposure or outcome | Sample size | Ancestry | Links for data | PMID |  |
| Circulating levels of 41 cytokines and growth factors | 8,293 participants | European ancestry | http://computationalmedicine.fi/data# | 27989323 |  |
|  |  |  | Cytokine_GWAS |  |  |
| Inflammatory bowel disease (discovery) | 12,882 cases, 21,770 controls | European ancestry | [https://gwas.mrcieu.ac.uk/datasets/.](https://gwas.mrcieu.ac.uk/datasets/) |  |  |
| Ulcerative colitis | 6,968 cases, 20,464 controls |  |  | 26192919 |  |
| Crohn's disease | 5,956 cases, 14,927 controls |  |  |  |  |
| Inflammatory bowel disease (validation) | 7,045 cases, 449,282 controls | UK biobank | [https://cnsgenomics.com/content/data.](https://cnsgenomics.com/content/data.) | 33608531 |  |

**Supplementary Table 2** Details of the numbers of genetic instruments, variance explained by the selected instruments and F-statistic for each cytokine and growth factor.

| Cytokines/ Growth factors | Abbreviation | No. Of SNP | Variance explained the primary instrumental variables* | F-statistic median (range)¶ | Power |
| --- | --- | --- | --- | --- | --- |
| Beta nerve growth factor | β-NGF | 1 | 0.011 | 36.50 | 0.225 |
| Cutaneous T-cell attracting (CCL27) | CTACK | 4 | 0.074 | 44.70(29.88-142.66) | 0.112 |
| Eotaxin (CCL11) | Eotaxin | 3 | 0.014 | 34.86(32.52-39.94) | 0.064 |
| Basic fibroblast growth factor | FGF-basic | 0 | \ | \ | \ |
| Granulocyte colony-stimulating factor | G-CSF | 0 | \ | \ | \ |
| Growth regulated oncogene-α (CXCL1) | GRO-a | 1 | 0.052 | 184.38 | 0.091 |
| Hepatocyte growth factor | HGF | 2 | 0.015 | 49.03(40.82-57.25) | 0.041 |
| Interferon-gamma | IFN-γ | 1 | 0.002 | 32.34 | 0.092 |
| Interleukin-1 receptor antagonist | IL-1rα | 0 | [\](file:///C:\\" \o "file:///C:") | \ | \ |
| Interleukin-1-beta | IL-1β | 0 | \ | \ | \ |
| Interleukin-2 | IL-2 | 0 | \ | \ | \ |
| Interleukin-2 receptor, alpha subunit | IL-2rα | 1 | 0.097 | 167.61 | 0.080 |
| Interleukin-4 | IL-4 | 0 | \ | \ | \ |
| Interleukin-5 | IL-5 | 1 | 0.011 | 37.93 | 0.067 |
| Interleukin-6 | IL-6 | 0 | \ | \ | \ |
| Interleukin-7 | IL-7 | 1 | 0.052 | 169.84 | 0.060 |
| Interleukin-8 (CXCL8) | IL-8 | 0 | \ | \ | \ |
| Interleukin-9 | IL-9 | 0 | \ | \ | \ |
| Interleukin-10 | IL-10 | 1 | 0.005 | 37.50 | 0.119 |
| Interleukin-12p70 | IL-12p70 | 5 | 0.022 | 38.42(32.26-50.14) | 0.075 |
| Interleukin-13 | IL-13 | 2 | 0.093 | 161.48(30.12-292.85) | 0.046 |
| Interleukin-16 | IL-16 | 3 | 0.092 | 40.59(31.05-131.98) | 0.046 |
| Interleukin-17 | IL-17 | 1 | 0.006 | 38.97 | 0.831 |
| Interleukin-18 | IL-18 | 5 | 0.076 | 45.41(31.75-96.17) | 0.858 |
| Interferon gamma-induced protein 10 (CXCL10) | IP-10 | 2 | 0.019 | 31.58(31.11-32.04) | 0.073 |
| Monocyte chemotactic protein-1 (CCL2) | MCP-1 | 3 | 0.029 | 45.41(33.50-91.78) | 0.156 |
| Monocyte specific chemokine 3 (CCL7) | MCP-3 | 0 | [\](file:///C:\\" \o "file:///C:") | \ | \ |
| Macrophage colony-stimulating factor | M-CSF | 1 | 0.016 | 31.64 | 0.139 |
| Macrophage migration inhibitory factor (glycosylation-inhibiting factor) | MIF | 1 | 0.011 | 39.05 | 0.167 |
| Monokine induced by interferon-gamma (CXCL9) | MIG | 1 | 0.010 | 42.38 | 0.984 |
| Macrophage inflammatory protein-1α (CCL3) | MIP-1α | 0 | \ | \ | \ |
| Macrophage inflammatory protein-1β (CCL4) | MIP-1b^§^ | 37 | 0.427 | 53.10(31.08-789.15) | 0.818 |
| Platelet derived growth factor BB | PDGF-bb | 7 | 0.076 | 47.74(29.91-245.35) | 0.072 |
| Regulated on activation, normal T Cell expressed and secreted (CCL5) | RANTES | 1 | 0.004 | 29.99 | 0.028 |
| Stem cell factor | SCF | 2 | 0.010 | 40.24(31.81-48.67) | 0.038 |
| Stem cell growth factor beta | SCGF-β | 6 | 0.107 | 59.31(35.25-99.38) | 0.138 |
| Stromal cell-derived factor-1 alpha (CXCL12) | SDF-1α | 0 | \ | \ | \ |
| Tumor necrosis factor-alpha | TNF-α | 0 | \ | \ | \ |
| Tumor necrosis factor-beta | TNF-β | 2 | 0.104 | 65.44(50.93-79.96) | 0.048 |
| TNF-related apoptosis inducing ligand | TRAIL | 14 | 0.256 | 120.60(30.12-370.00) | 0.269 |
| Vascular endothelial growth factor | VEGF | 11 | 0.182 | 46.15(32.77-784.00) | 0.609 |

Abbreviations: No., number; SNP, single nucleotide polymorphism.

* β: effect size estimates of the SNPs of cytokines/growth factors levels.; MAF: minimum allele frequency. The variance of each cytokine was calculated by using an additive model under the assumption of no interaction between each SNPs. (Georgakis et al. Circulation, 2019, 139(2): 256-268.)

¶ F= β^2^ / se^2^. β: effect size estimate of SNP. se: standard error of SNP of cytokines/growth factors. (Li & Martin Comput Stat Data Anal, 2002,40, 21-26).

**Supplementary Table 3** Details of the genetic variants with potential pleiotropy among instrumental variables used for inflammatory bowel disease, Crohn's Disease and ulcerative colitis.

| Trait | SNP | Pleiotropic Trait^*^ | *P*-value | PMID |
| --- | --- | --- | --- | --- |
| IBD | rs10737481 | Rheumatoid arthritis,ulcerative colitis | 3E-19 | 33686288 |
|  | rs10800314 | Ankylosing spondylitis, psoriasis, sclerosing cholangitis | 1E-32 | 26974007 |
|  | rs11209026 | Rheumatoid arthritis,ulcerative colitis | 3E-13 | 33686288 |
|  |  | Ankylosing spondylitis | 6E-28 | 23749187 |
|  |  | Psoriasis | 2E-10 | 25574825 |
|  |  | Interleukin 23 receptor measurement | 3E-16 | 28240269 |
|  | rs3024493 | Systemic lupus erythematosus | 2E-13 | 28714469 |
|  |  | Type2 diabetes mellitus | 5E-10 | 30572963 |
|  |  | Rheumatoid arthritis,ulcerative colitis | 1E-10 | 33686288 |
|  |  | Ankylosing spondylitis, psoriasis, sclerosing cholangitis | 1E-38 | 26974007 |
|  | rs34920465 | Ankylosing spondylitis, psoriasis, sclerosing cholangitis | 2E-13 | 26974007 |
|  |  | Bonedensity | 5E-10 | 29304378 |
|  |  | Hematocrit | 1E-10 | 32888494 |
|  |  | Hemoglobin measurement | 1E-38 | 32888494 |
|  | rs112694524 | Appendicular lean mass | 3E-11 | 31761296 |
|  |  | Intraocular pressure measurement | 1E-10 | 32352494 |
|  |  | Reticulocyte measurement | 4E-19 | 32888494 |
|  |  | BMI-adjustedwaist-hipratio | 1E-8 | 34021172 |
|  |  | Neutrophil count | 2E-17 | 34594039 |
|  |  | Creatinine measurement | 1E-23 | 34594039 |
|  | rs11677953 | Heel bone mineral density | 6E-10 | 30595370 |
|  | rs2241878 | Alkaline phosphatase measurement | 3E-13 | 33972514 |
|  |  | Eosinophil count | 2E-10 | 32888493 |
|  |  | Ankylosing spondylitis | 1E-8 | 23749187 |
|  | rs13063312 | Hepatocyte growth factor-likeprotein measurement | 4E-30 | 28240269 |
|  | rs1873625 | Self reported educational attainment | 2E-95 | 30038396 |
|  |  | Mathematical ability | 5E-30 | 30038396 |
|  | rs10045431 | Obsolete_interleukin12 receptorsubunitbeta-1 measurement | 9E-17 | 25147954 |
|  |  | Blood protein measurement | 1E-10 | 27532455 |
|  |  | Rheumatoid arthritis,Crohn's disease | 1E-8 | 33686288 |
|  |  | Interleukin-23 measurement | 8E-29 | 34814699 |
|  | rs139746351 | Mosquito bite reaction it chintensity measurement | 3E-14 | 28199695 |
|  | rs254559 | High density lipoprotein cholesterol measurement | 2E-11 | 34887591 |
|  |  | ApolipoproteinA1 measurement | 2E-17 | 32203549 |
|  | rs35260072 | Mosquito bite reaction it chintensity measurement | 8E-38 | 28199695 |
|  |  | Hstidine betaine(hercynine) measurement | 3E-11 | 36635386 |
|  | rs6880778 | Multiple sclerosis | 8E-20 | 24076602 |
|  | rs144515162 | Interleukin-21 measurement | 1E-13 | 29875488 |
|  |  | Complement C4 measurement | 1E-30 | 29875488 |
|  |  | Hepatocyte nuclear factor 4-alpha measurement | 3E-13 | 29875488 |
|  |  | Ubiquitin carbo×yl-terminal hydrolase25 measurement | 8E-12 | 29875488 |
|  | rs148844907 | Susceptibility to Mycobacterium tuberculosis infection measurement | 2E-12 | 28928442 |
|  | rs2230365 | Age at menopause | 8E-10 | 26414677 |
|  |  | Monocyte count | 7E-16 | 34469753 |
|  |  | BMI-adjusted hip circumference | 1E-12 | 34021172 |
|  | rs444210 | Lung adenocarcinoma | 5.00E-08 | 28604730 |
|  | rs4712528 | Psoriasis vulgaris | 8E-11 | 26626624 |
|  | rs6927172 | Childhood onset asthma | 3E-13 | 30929738 |
|  |  | Asthma | 6E-11 | 32296059 |
|  | rs9370774 | Self reported educational attainment | 8E-18 | 30038396 |
|  | rs1551399 | Ankylosing spondylitis, psoriasis, sclerosing cholangitis | 4E-19 | 26974007 |
|  | rs1887428 | Neutrophil count | 2E-12 | 32888494 |
|  |  | Systemic lupuseryth ematosus | 2E-17 | 27399966 |
|  |  | Erythrocyte count | 1E-16 | 32888494 |
|  |  | Leukocyte count | 7E-12 | 32888494 |
|  |  | Monocyt epercent age ofleukocytes | 2E-10 | 32888494 |
|  |  | Mean corpuscular hemoglobin | 1E-14 | 32888494 |
|  |  | Mean corpuscularvolume | 2E-13 | 32888494 |
|  | rs4077515 | IGA glomerulo nephritis | 1E-8 | 25305756 |
|  |  | Protein measurement | 2E-35 | 34648354 |
|  | rs4246905 | Autoimmune thyroid disease, systemicl upuseryth ematosus, type1 diabetes mellitus, ankylosing spondylitis, | 1.00E-08 | 26301688 |
|  | rs1250573 | Ankylosing spondylitis, psoriasis, sclerosing cholangitis | 2E-22 | 26974007 |
|  | rs6584283 | Colorectal cancer | 6E-11 | 31826910 |
|  |  | Rheumatoida rthritis,ulcerative colitis | 3E-10 | 33686288 |
|  |  | Monocyte count | 1E-9 | 29403010 |
|  | rs11236797 | Allergic rhinitis | 5E-32 | 31361310 |
|  |  | Antihistamine use measurement | 1E-13 | 31015401 |
|  |  | Ankylosing spondylitis,psoriasis,sclerosingc holangitis | 3E-43 | 26974007 |
|  |  | Childhood onset asthma | 1E-93 | 30929738 |
|  |  | Asthma | 6E-62 | 30929738 |
|  | rs56062135 | Asthma | 3E-23 | 29785011 |
|  |  | Childhood onset asthma | 3E-33 | 31669095 |
|  |  | Adult onset asthma | 6E-12 | 31036433 |
|  |  | Eosinophilic esophagitis | 4E-10 | 34506852 |
|  |  | Thyroid carcinoma | 5E-8 | 28195142 |
|  |  | Asthma,endometriosis | 7E-22 | 35472084 |
|  |  | Allergic disease | 2E-9 | 32603359 |
|  |  | Coronary artery disease | 2E-22 | 36474045 |
|  |  | Nasal disorder | 7E-10 | 36653354 |
|  |  | Chronic rhinosinusitis | 1E-8 | 36653354 |
|  | rs11548656 | Blood protein measurement | 1E-10 | 32929287 |
|  |  | Non-albumin protein levels | 1E-11 | 33462484 |
|  | rs12446550 | Intelligence | 2E-19 | 29326435 |
|  |  | Birthweight, parental genotype effect measurement | 5E-8 | 31043758 |
|  |  | Alcohol consumption measurement | 1E-31 | 36477530 |
|  | rs72798422 | Oral ulcer | 3E-22 | 30837455 |
|  | rs12936409 | Rheumatoid arthritis | 1E-10 | 24532676 |
|  | rs744166 | Multiple sclerosis | 3E-10 | 20159113 |
|  | rs2542147 | Atopic eczema | 3E-26 | 37794016 |
|  | rs10408351 | Reticulocyte measurement | 6E-18 | 32888494 |
|  | rs142770866 | Leukocyte count | 1E-13 | 32888494 |
|  |  | COVID-19 | 9E-21 | 37198478 |
|  | rs6062496 | Ankylosing spondylitis, psoriasis, sclerosing cholangitis | 2E-30 | 26974007 |
|  | rs2836882 | C-reactive protein measurement | 6E-45 | 31900758 |
|  |  | Alkaline phosphatase measurement | 1E-8 | 33547301 |
|  |  | Autoimmune thyroid disease,systemic lupuseryth ematosus,type1 diabetes mellitus | 5E-8 | 26301688 |
|  |  | Hemoglobin measurement | 2E-9 | 27863252 |
|  | rs1003342 | Rheumatoid arthritis,Crohn'sd isease | 6E-10 | 33686288 |
|  |  | Lymphocyte count | 4E-23 | 34469753 |
|  |  | Platelet-to-lymphocyte ratio | 7E-20 | 34469753 |
|  |  | Cup-to-discratio measurement | 4E-27 | 34077760 |
|  | rs131657 | Mean corpuscular volume | 5E-77 | 32888494 |
| CD | rs11209026 | Ankylosing spondylitis | 6E-28 | 23749187 |
|  |  | Psoriasis | 2E-10 | 25574825 |
|  |  | Rheumatoid arthritis, ulcerative colitis | 3E-13 | 33686288 |
|  |  | Interleukin 23 receptor measurement | 3E-16 | 28240269 |
| CD | rs3024505 | Type 1 diabetes mellitus | 5E-10 | 21829393 |
|  |  | Systemic lupus erythematosus | 2E-18 | 36750564 |
| CD | rs7423615 | Eosinophil count | 1E-21 | 32888493 |
|  |  | Eosinophil percentage of leukocytes | 2E-19 | 32888494 |
| CD | rs78487399 | Myeloid white cell count | 3E-10 | 27863252 |
|  |  | Neutrophil count, basophil count | 2E-11 | 27863252 |
|  |  | Granulocyte count | 3E-11 | 27863252 |
| CD | rs1873625 | Self reported educational attainment | 2E-95 | 30038396 |
|  |  | Mathematical ability | 5E-30 | 30038396 |
| CD | rs13135092 | Alcohol consumption measurement | 2E-35 | 32451486 |
|  |  | Bain volume measurement | 2E-20 | 33875891 |
|  |  | Brain age measurement | 1E-13 | 36707530 |
|  |  | Bipolar disorder | 1E-8 | 32606422 |
|  |  | Bitter alcoholic beverage consumption measurement | 3E-14 | 31046077 |
|  |  | Back pain | 3E-8 | 30747904 |
|  |  | Cortical thickness | 3E-15 | 33875891 |
|  |  | Cognitive function measurement | 3E-10 | 32895543 |
|  |  | Cerebellar volume measurement | 3E-22 | 35079123 |
|  |  | Ceramide measurement | 2E-9 | 35668104 |
|  |  | Diet measurement | 7E-10 | 34426670 |
|  |  | Educational attainment | 4E-58 | 35361970 |
|  |  | Fornix volume measurement | 1E-18 | 36707530 |
|  |  | Grip strength measurement | 3E-26 | 29691431 |
|  |  | HDL cholesterol change measurement,physical activity | 5E-19 | 30670697 |
|  |  | High density lipoprotein cholesterol measurement | 7E-21 | 29507422 |
|  |  | Insomnia measurement | 3E-16 | 30804565 |
|  |  | Leukocyte count | 4E-8 | 30595370 |
|  |  | Low density lipoprotein cholesterol measurement | 3E-8 | 29507422 |
|  |  | Multisite chronic pain | 3E-14 | 33830993 |
|  |  | Obese body mass index status | 1E-9 | 30677029 |
|  |  | Opioid use disorder | 2E-12 | 37156939 |
|  |  | Risk-taking behaviour | 3E-11 | 30643258 |
|  |  | Schizophrenia | 9E-13 | 33479212 |
|  |  | Schizophrenia,anorexia nervosa | 6E-9 | 33686288 |
|  |  | Serum albumin measurement | 3E-13 | 34321204 |
|  |  | Sexual dimorphism measurement | 3E-9 | 37277458 |
|  |  | Taste liking measurement | 8E-13 | 35585065 |
|  |  | Total cholesterol measurement | 5E-11 | 29507422 |
|  |  | Verbal-numerical reasoning measurement | 8E-17 | 32895543 |
|  |  | Visceral adipose tissue measurement | 7E-13 | 31501611 |
| CD | rs10045431 | Obsolete_interleukin 12 receptor subunit beta-1 measurement | 9E-17 | 25147954 |
|  |  | Blood protein measurement | 1E-10 | 27532455 |
|  |  | Rheumatoid arthritis, Crohn's disease | 1E-9 | 33686288 |
|  |  | Interleukin-23 measurement | 8E-29 | 34814699 |
| CD | rs10055349 | Erythrocyte count | 5E-9 | 34594039 |
| CD | rs12717899 | Mean corpuscular volume | 8E-10 | 34594039 |
| CD | rs139746351 | Mosquito bite reaction itch intensity measurement | 3E-14 | 28199695 |
| CD | rs147018773 | Rheumatoid arthritis, Crohn's disease | 2E-9 | 33686288 |
| CD | rs2188962 | Asthma, cardiovascular disease | 2E-9 | 35126453 |
|  |  | Diastolic blood pressure | 3E-11 | 27618448 |
| CD | rs148844907 | Susceptibility to Mycobacterium tuberculosis infection measurement | 2E-12 | 28928442 |
| CD | rs2856997 | BMI-adjusted hip circumference | 9E-9 | 34021172 |
| CD | rs28701841 | Ankylosing spondylitis, psoriasis, sclerosing cholangitis | 6E-10 | 26974007 |
| CD | rs1456896 | Leukocyte count | 6E-23 | 30595370 |
|  |  | Myeloid white cell count | 2E-18 | 32888493 |
|  |  | Neutrophil count | 3E-25 | 34594039 |
| CD | rs1887428 | Systemic lupus erythematosus | 2E-17 | 27399966 |
|  |  | Neutrophil count | 2E-12 | 32888494 |
|  |  | Erythrocyte count | 1E-16 | 32888494 |
|  |  | Leukocyte count | 7E-12 | 32888494 |
|  |  | Monocyte percentage of leukocytes | 2E-10 | 32888494 |
|  |  | Mean corpuscular hemoglobin | 1E-14 | 32888494 |
| CD | rs4077515 | IGA glomerulonephritis | 1E-9 | 25305756 |
|  |  | Protein measurement | 2E-35 | 34648354 |
| CD | rs10761659 | rheumatoid arthritis, Crohn's disease | 2E-20 | 33686288 |
| CD | rs1250573 | Ankylosing spondylitis, psoriasis, sclerosing cholangitis | 2E-22 | 26974007 |
| CD | rs1332099 | Rheumatoid arthritis, Crohn's disease | 5E-10 | 33686288 |
|  |  | Autoimmune thyroid disease, | 9E-11 | 26301688 |
| CD | rs11236797 | Childhood onset asthma | 1E-93 | 30929738 |
|  |  | Allergic rhinitis | 5E-32 | 31361310 |
|  |  | Antihistamine use measurement | 1E-13 | 31015401 |
|  |  | Ankylosing spondylitis, psoriasis, sclerosing cholangitis | 3E-43 | 26974007 |
|  |  | Asthma, age at onset | 7E-18 | 30929738 |
| CD | rs56062135 | Asthma | 3E-23 | 29785011 |
|  |  | Eosinophilic esophagitis | 4E-10 | 34506852 |
|  |  | Thyroid carcinoma | 5E-9 | 28195142 |
|  |  | Allergic disease | 2E-8 | 32603359 |
|  |  | Nasal disorder | 7E-10 | 36653354 |
|  |  | Chronic rhinosinusitis | 1E-8 | 36653354 |
|  |  | Coronary artery disease | 2E-22 | 36474045 |
| CD | rs2076756 | Rheumatoid arthritis | 5E-13 | 33686288 |
| CD | rs72798422 | Oral ulcer | 3E-22 | 30837455 |
| CD | rs12936409 | Rheumatoid arthritis | 1E-10 | 24532676 |
| CD | rs744166 | Multiple sclerosis | 3E-10 | 20159113 |
| CD | rs281379 | Body height | 5E-8 | 23449627 |
|  |  | Asthma exacerbation measurement | 3E-9 | 33328473 |
|  |  | Alcohol consumption measurement | 4E-21 | 30643251 |
|  |  | Blood protein measurement | 3E-16 | 35078996 |
|  |  | Taste liking measurement | 4E-11 | 35585065 |
|  |  | C-C motif chemokine 25 measurement | 4E-22 | 34648354 |
| CD | rs8178977 | Basal cell carcinoma | 3E-9 | 31174203 |
| CD | rs1056441 | Blood protein measurement | 2E-9 | 30072576 |
|  |  | C-reactive protein measurement | 4E-24 | 31900758 |
| CD | rs151314883 | Rheumatoid arthritis | 4E-11 | 33686288 |
| CD | rs8137950 | Mean corpuscular volume | 7E-23 | 29403010 |
| UC | rs10737481 | Rheumatoid arthritis | 3E-19 | 33686288 |
| UC | rs11209026 | Rheumatoid arthritis | 3E-13 | 33686288 |
|  |  | Akylosing spondylitis | 6E-28 | 23749187 |
|  |  | Psoriasis | 2E-10 | 25574825 |
|  |  | Interleukin 23 receptor measurement | 3E-16 | 28240269 |
| UC | rs1801274 | Systemic lupus erythematosus | 2E-14 | 28714469 |
|  |  | Myeloid white cell count | 3E-54 | 32929287 |
|  |  | Blood protein measurement | 1E-90 | 32929287 |
|  |  | Cerebrospinal fluid biomarker measurement | 9E-32 | 28031287 |
|  |  | Ankylosing spondylitis | 1E-91 | 23749187 |
|  |  | Mucocutaneous lymph node syndrome | 7E-11 | 22081228 |
|  |  | Basophil percentage of leukocytes | 2E-18 | 32888494 |
|  |  | Low affinity immunoglobulin gamma Fc region receptor II-b measurement | 4E-206 | 36168886 |
| UC | rs3024493 | Systemic lupus erythematosus | 2E-13 | 28714469 |
|  |  | Type 2 diabetes mellitus | 5E-10 | 30572963 |
|  |  | Rheumatoid arthritis, ulcerative colitis | 1E-10 | 33686288 |
|  |  | Ankylosing spondylitis, psoriasis,sclerosing cholangitis | 1E-38 | 26974007 |
| UC | rs34920465 | Ankylosing spondylitis, psoriasis,sclerosing cholangitis | 3E-13 | 26974007 |
|  |  | Bone density | 3E-35 | 29304378 |
|  |  | Hematocrit | 2E-14 | 32888494 |
|  |  | Hemoglobin measurement | 6E-16 | 32888494 |
| UC | rs4676410 | Sclerosing cholangitis | 2E-9 | 22821403 |
|  |  | Eosinophil count | 2E-10 | 32888493 |
|  |  | Ankylosing spondylitis | 1E-8 | 23749187 |
| UC | rs254559 | High density lipoprotein cholesterol measurement | 2E-11 | 34887591 |
|  |  | Apolipoprotein A 1 measurement | 2E-17 | 32203549 |
| UC | rs56167332 | Ankylosing spondylitis, sclerosing cholangitis | 3E-43 | 26974007 |
|  |  | Leprosy, Crohn's disease | 2E-17 | 35512355 |
| UC | rs144515162 | Interleukin-21 measurement | 1E-13 | 29875488 |
|  |  | Complement C4 measurement | 1E-30 | 29875488 |
|  |  | Hepatocyte nuclear factor 4-alpha measurement | 3E-13 | 29875488 |
|  |  | Ubiquitin carboxyl-terminal hydrolase 25 measurement | 8E-12 | 29875488 |
| UC | rs148844907 | Susceptibility to Mycobacterium tuberculosis infection measurement | 2E-12 | 28928442 |
| UC | rs17369215 | Cognitive function measurement | 2E-11 | 30038396 |
| UC | rs3095307 | BMI-adjusted hip circumference | 3E-9 | 34021172 |
| UC | rs6933404 | Primary biliary cirrhosis | 1E-10 | 26394269 |
| UC | rs798502 | BMI-adjusted waist circumference | 3E-11 | 25673412 |
| UC | rs1887428 | Systemic lupus erythematosus | 2E-17 | 27399966 |
|  |  | Neutrophil count | 2E-12 | 32888494 |
|  |  | Erythrocyte count | 1E-16 | 32888494 |
|  |  | Leukocyte count | 7E-12 | 32888494 |
|  |  | Monocyte percentage of leukocytes | 2E-10 | 32888494 |
|  |  | Mean corpuscular hemoglobin | 1E-14 | 32888494 |
|  |  | Mean corpuscular volume | 2E-13 | 32888494 |
| UC | rs2212434 | Food allergy measurement | 9E-11 | 29051540 |
|  |  | Atopic eczema | 5E-13 | 26482879 |
| UC | rs12817473 | Rheumatoid arthritis | 5E-8 | 33686288 |
| UC | rs6062496 | Ankylosing spondylitis, psoriasis, sclerosing cholangitis | 2E-30 | 26974007 |
| UC | rs9977672 | Mean corpuscular hemoglobin | 5E-11 | 30595370 |
|  |  | Platelet crit | 1E-8 | 32888494 |
|  |  | Neutrophil count,basophil count | 7E-10 | 27863252 |
|  |  | Ankylosingspondylitis, psoriasis, sclerosingcholangitis | 5E-54 | 26974007 |
|  |  | Neutrophil count,eosinophil count | 5E-9 | 27863252 |
|  |  | Granulocyte count | 4E-8 | 27863252 |
|  |  | Leukocyte count | 3E-18 | 32888494 |
|  |  | Obsolete_red blood cell distribution width | 5E-13 | 32888493 |
|  |  | Mean corpuscular hemoglobin concentration | 2E-13 | 32888493 |
|  |  | Neutrophil count | 9E-25 | 32888493 |
|  |  | Myeloid white cell count | 2E-26 | 32888493 |

Abbreviations: CD, Crohn's Disease; IBD, inflammatory Bowel Disease; SE, standard error; SNP, single nucleotide polymorphism; UC, ulcerative colitis. ^*^ From the GWAS Catalog (http://www.ebi.ac.uk/gwas, last accessed on November 23th, 2023).

**Supplementary Table 4** Characteristics of the genetic variants associated with the inflammatory bowel disease, Crohn's disease and ulcerative colitis.

| Trait | SNP | Chr | Position | Effect allele | Beta | SE | *P*-value | R^2^ |
| --- | --- | --- | --- | --- | --- | --- | --- | --- |
| IBD | rs10917547 | 1 | 20143142 | A | 0.100 | 0.018 | 1.89E-08 | 0.001 |
| IBD | rs112874012 | 1 | 67674756 | T | -0.250 | 0.045 | 2.60E-08 | 0.001 |
| IBD | rs1886731 | 1 | 2472081 | T | 0.097 | 0.018 | 3.08E-08 | 0.001 |
| IBD | rs35730213 | 1 | 200874229 | C | -0.151 | 0.019 | 6.91E-15 | 0.002 |
| IBD | rs7523335 | 1 | 8180210 | A | -0.141 | 0.023 | 4.16E-10 | 0.001 |
| IBD | rs112401990 | 2 | 61199327 | A | 0.142 | 0.017 | 2.84E-16 | 0.002 |
| IBD | rs4851586 | 2 | 103064264 | T | 0.122 | 0.019 | 2.33E-10 | 0.001 |
| IBD | rs45528737 | 4 | 123275555 | T | 0.167 | 0.030 | 2.66E-08 | 0.001 |
| IBD | rs6826501 | 4 | 36076676 | T | -0.093 | 0.017 | 4.12E-08 | 0.001 |
| IBD | rs113825849 | 5 | 40294919 | C | -0.584 | 0.080 | 3.10E-13 | 0.002 |
| IBD | rs11949375 | 5 | 150202980 | T | -0.189 | 0.028 | 3.00E-11 | 0.001 |
| IBD | rs17454191 | 5 | 158827497 | T | 0.134 | 0.018 | 2.06E-13 | 0.002 |
| IBD | rs6873866 | 5 | 96247810 | T | 0.107 | 0.018 | 1.09E-09 | 0.001 |
| IBD | rs114607072 | 6 | 31351940 | T | 0.290 | 0.050 | 6.07E-09 | 0.001 |
| IBD | rs115308106 | 6 | 32683666 | A | 0.400 | 0.067 | 2.31E-09 | 0.001 |
| IBD | rs115312361 | 6 | 32488215 | A | 0.184 | 0.023 | 4.95E-16 | 0.002 |
| IBD | rs116465569 | 6 | 32536986 | T | 0.201 | 0.021 | 9.27E-23 | 0.003 |
| IBD | rs117292830 | 6 | 31218268 | A | 0.430 | 0.058 | 1.42E-13 | 0.002 |
| IBD | rs28383456 | 6 | 32609453 | T | -0.178 | 0.020 | 5.77E-19 | 0.002 |
| IBD | rs34190331 | 6 | 111840820 | A | 0.177 | 0.030 | 5.39E-09 | 0.001 |
| IBD | rs6911490 | 6 | 106522027 | T | 0.143 | 0.021 | 6.82E-12 | 0.001 |
| IBD | rs75565243 | 6 | 31463379 | A | 0.349 | 0.055 | 1.55E-10 | 0.001 |
| IBD | rs9272514 | 6 | 32606385 | T | -0.235 | 0.021 | 1.59E-29 | 0.004 |
| IBD | rs4730272 | 7 | 107478227 | A | 0.134 | 0.018 | 4.50E-14 | 0.002 |
| IBD | rs7797798 | 7 | 107447051 | C | 0.096 | 0.017 | 2.95E-08 | 0.001 |
| IBD | rs10761659 | 10 | 64445564 | A | -0.162 | 0.017 | 4.07E-21 | 0.003 |
| IBD | rs10826797 | 10 | 30690376 | T | -0.102 | 0.019 | 3.47E-08 | 0.001 |
| IBD | rs12764283 | 10 | 35530460 | A | 0.127 | 0.018 | 1.57E-12 | 0.001 |
| IBD | rs140892874 | 12 | 40824798 | T | -0.410 | 0.051 | 1.28E-15 | 0.002 |
| IBD | rs2193041 | 12 | 68502110 | A | -0.134 | 0.017 | 6.91E-15 | 0.002 |
| IBD | rs3850378 | 14 | 88417517 | T | -0.155 | 0.028 | 3.80E-08 | 0.001 |
| IBD | rs145126485 | 16 | 50918662 | A | -0.283 | 0.050 | 1.40E-08 | 0.001 |
| IBD | rs2076756 | 16 | 50756881 | A | -0.188 | 0.019 | 5.59E-24 | 0.003 |
| IBD | rs9934775 | 16 | 50383077 | T | -0.140 | 0.023 | 1.72E-09 | 0.001 |
| IBD | rs3091316 | 17 | 32593974 | A | -0.112 | 0.019 | 3.59E-09 | 0.001 |
| IBD | rs2129944 | 19 | 10516198 | T | 0.119 | 0.020 | 1.57E-09 | 0.001 |
| IBD | rs4807569 | 19 | 1123378 | A | -0.139 | 0.021 | 2.37E-11 | 0.001 |
| IBD | rs1736161 | 21 | 16833222 | A | -0.123 | 0.017 | 1.34E-12 | 0.001 |
| IBD | rs7282490 | 21 | 45615741 | A | -0.145 | 0.017 | 1.28E-17 | 0.002 |
| IBD | rs7285952 | 22 | 39733096 | T | 0.176 | 0.024 | 7.60E-14 | 0.002 |
| CD | rs6588243 | 1 | 67603383 | A | -0.132 | 0.023 | 1.78E-08 | 0.002 |
| CD | rs6704109 | 1 | 172857050 | T | 0.202 | 0.026 | 2.77E-15 | 0.003 |
| CD | rs697693 | 1 | 7886424 | A | 0.172 | 0.028 | 8.36E-10 | 0.002 |
| CD | rs7543234 | 1 | 155253308 | T | 0.155 | 0.027 | 6.10E-09 | 0.002 |
| CD | rs112401990 | 2 | 61199327 | A | 0.132 | 0.024 | 2.35E-08 | 0.001 |
| CD | rs12692254 | 2 | 234161211 | A | -0.301 | 0.023 | 1.86E-38 | 0.008 |
| CD | rs4851586 | 2 | 103064264 | T | 0.169 | 0.026 | 9.94E-11 | 0.002 |
| CD | rs2013718 | 5 | 158828480 | A | 0.175 | 0.025 | 4.20E-12 | 0.002 |
| CD | rs6873866 | 5 | 96247810 | T | 0.168 | 0.024 | 2.07E-12 | 0.002 |
| CD | rs7713270 | 5 | 40440063 | T | 0.297 | 0.024 | 6.97E-35 | 0.007 |
| CD | rs114607072 | 6 | 31351940 | T | 0.442 | 0.063 | 2.20E-12 | 0.002 |
| CD | rs12194825 | 6 | 20835260 | A | -0.172 | 0.030 | 8.00E-09 | 0.002 |
| CD | rs143345302 | 6 | 31226665 | T | 0.212 | 0.036 | 3.44E-09 | 0.002 |
| CD | rs444210 | 6 | 167390242 | A | -0.163 | 0.023 | 1.03E-12 | 0.002 |
| CD | rs921720 | 8 | 126534671 | A | -0.163 | 0.024 | 6.40E-12 | 0.002 |
| CD | rs3810936 | 9 | 117552885 | T | -0.208 | 0.026 | 2.46E-15 | 0.003 |
| CD | rs2505640 | 10 | 35459497 | A | 0.146 | 0.024 | 7.61E-10 | 0.002 |
| CD | rs11564236 | 12 | 40828306 | A | -0.519 | 0.060 | 2.85E-18 | 0.004 |
| CD | rs1932990 | 13 | 44460242 | T | 0.153 | 0.026 | 6.02E-09 | 0.002 |
| CD | rs4902642 | 14 | 69210199 | A | -0.129 | 0.024 | 4.34E-08 | 0.001 |
| CD | rs111564463 | 16 | 50508185 | A | -0.563 | 0.093 | 1.51E-09 | 0.002 |
| CD | rs145126485 | 16 | 50918662 | A | -0.627 | 0.062 | 2.26E-24 | 0.005 |
| CD | rs147684209 | 16 | 28867061 | T | -0.155 | 0.024 | 2.34E-10 | 0.002 |
| CD | rs4486887 | 16 | 50677571 | T | -0.196 | 0.025 | 2.04E-15 | 0.003 |
| CD | rs7499231 | 16 | 50427128 | A | 0.239 | 0.042 | 1.16E-08 | 0.002 |
| CD | rs76532080 | 16 | 50488249 | T | 0.358 | 0.052 | 6.48E-12 | 0.002 |
| CD | rs3091315 | 17 | 32593665 | A | 0.180 | 0.026 | 9.52E-12 | 0.002 |
| CD | rs80262450 | 18 | 12818922 | A | 0.283 | 0.035 | 1.08E-15 | 0.003 |
| CD | rs2129944 | 19 | 10516198 | T | 0.156 | 0.027 | 7.81E-09 | 0.002 |
| CD | rs74179925 | 19 | 10567208 | A | 0.173 | 0.030 | 8.83E-09 | 0.002 |
| CD | rs1297271 | 21 | 16823163 | T | -0.155 | 0.024 | 6.28E-11 | 0.002 |
| CD | rs7276302 | 21 | 45614159 | A | 0.172 | 0.023 | 1.23E-13 | 0.003 |
| UC | rs10917547 | 1 | 20143142 | A | 0.173 | 0.022 | 5.51E-15 | 0.002 |
| UC | rs1317209 | 1 | 20140036 | A | 0.146 | 0.027 | 3.47E-08 | 0.001 |
| UC | rs1886731 | 1 | 2472081 | T | 0.141 | 0.022 | 2.25E-10 | 0.001 |
| UC | rs35730213 | 1 | 200874229 | C | -0.167 | 0.025 | 8.82E-12 | 0.002 |
| UC | rs7523335 | 1 | 8180210 | A | -0.170 | 0.029 | 2.29E-09 | 0.001 |
| UC | rs10182512 | 2 | 61189469 | A | 0.161 | 0.022 | 5.19E-13 | 0.002 |
| UC | rs12612675 | 2 | 219133137 | A | -0.123 | 0.022 | 1.98E-08 | 0.001 |
| UC | rs2276850 | 3 | 48669648 | A | 0.197 | 0.033 | 2.87E-09 | 0.001 |
| UC | rs9823546 | 3 | 49705512 | A | 0.177 | 0.022 | 2.29E-15 | 0.002 |
| UC | rs114152040 | 5 | 40444986 | A | 0.340 | 0.062 | 4.95E-08 | 0.001 |
| UC | rs115308106 | 6 | 32683666 | A | 0.450 | 0.081 | 2.77E-08 | 0.001 |
| UC | rs115312361 | 6 | 32488215 | A | 0.324 | 0.029 | 9.44E-29 | 0.004 |
| UC | rs117292830 | 6 | 31218268 | A | 0.615 | 0.070 | 2.20E-18 | 0.003 |
| UC | rs143210366 | 6 | 31492353 | T | -0.390 | 0.057 | 8.24E-12 | 0.002 |
| UC | rs144582178 | 6 | 32481800 | T | 0.276 | 0.028 | 4.06E-23 | 0.004 |
| UC | rs183231933 | 6 | 32524320 | T | -0.371 | 0.031 | 1.71E-32 | 0.005 |
| UC | rs28383456 | 6 | 32609453 | T | -0.337 | 0.026 | 1.07E-39 | 0.006 |
| UC | rs45627734 | 6 | 31474884 | A | 0.406 | 0.063 | 1.47E-10 | 0.001 |
| UC | rs7752873 | 6 | 106579332 | T | 0.182 | 0.030 | 1.83E-09 | 0.001 |
| UC | rs9272514 | 6 | 32606385 | T | -0.402 | 0.027 | 4.00E-51 | 0.008 |
| UC | rs10272963 | 7 | 107486902 | T | -0.172 | 0.022 | 1.69E-15 | 0.002 |
| UC | rs2301989 | 7 | 107443871 | A | -0.141 | 0.022 | 8.55E-11 | 0.002 |
| UC | rs3829111 | 9 | 139269483 | A | 0.156 | 0.021 | 2.89E-13 | 0.002 |
| UC | rs4574921 | 9 | 117538334 | T | 0.151 | 0.026 | 4.24E-09 | 0.001 |
| UC | rs7911680 | 10 | 101293468 | A | 0.172 | 0.021 | 8.27E-16 | 0.002 |
| UC | rs483905 | 11 | 96023427 | A | 0.129 | 0.023 | 1.57E-08 | 0.001 |
| UC | rs484356 | 11 | 114406639 | C | 0.134 | 0.023 | 3.95E-09 | 0.001 |
| UC | rs1359946 | 13 | 27536972 | A | 0.158 | 0.027 | 3.84E-09 | 0.001 |
| UC | rs9891174 | 17 | 38031802 | A | 0.145 | 0.021 | 7.17E-12 | 0.002 |
| UC | rs6017342 | 20 | 43065028 | A | -0.191 | 0.024 | 1.39E-15 | 0.002 |
| UC | rs137845 | 22 | 50439430 | A | -0.118 | 0.021 | 2.38E-08 | 0.001 |

Abbreviations: Chr, chromosome; CD, Crohn's Disease; IBD, inflammatory bowel disease; SE, standard error; SNP, single nucleotide polymorphism; UC, ulcerative colitis.

**Supplementary Table 5** Characteristics of instrumental variables used for circulating levels of 28 cytokines in this study.

| Cytokines/ Growth factors | SNP | Chr | Position | Effect allele | Beta | SE | *P*-value |
| --- | --- | --- | --- | --- | --- | --- | --- |
| β-NGF | rs28637706 | 19 | 34285368 | G | 0.1589 | 0.0263 | 1.42E-09 |
| CTACK | rs2070074 | 9 | 34649442 | G | -0.4467 | 0.0374 | 1.79E-32 |
| CTACK | rs58704839 | 9 | 34680554 | G | -0.1785 | 0.0284 | 3.29E-10 |
| CTACK | rs55764737 | 15 | 61323414 | C | -0.5313 | 0.0972 | 4.62E-08 |
| CTACK | rs135564 | 22 | 46535934 | G | 0.1893 | 0.0268 | 2.43E-12 |
| Eotaxin | rs342511 | 3 | 42578509 | G | -0.0927 | 0.0157 | 3.60E-09 |
| Eotaxin | rs3091309 | 3 | 46303184 | G | -0.1283 | 0.0203 | 3.63E-10 |
| Eotaxin | rs2024050 | 7 | 75460393 | G | -0.1728 | 0.0303 | 1.10E-08 |
| GRO-a | rs508977 | 4 | 74762383 | G | 0.3802 | 0.028 | 7.56E-42 |
| HGF | rs3748034 | 4 | 3446091 | G | -0.1495 | 0.0234 | 1.81E-10 |
| HGF | rs5745687 | 7 | 81359051 | C | 0.3072 | 0.0406 | 2.75E-14 |
| IFN-γ | rs45498698 | 1 | 22895820 | a | -0.3844 | 0.0676 | 1.13E-08 |
| IL-2rα | rs12722497 | 10 | 6095928 | C | -0.6279 | 0.0485 | 1.57E-38 |
| IL5 | rs7767396 | 6 | 43927050 | G | -0.1515 | 0.0246 | 7.69E-10 |
| IL7 | rs4320361 | 6 | 43928511 | G | 0.3245 | 0.0249 | 6.87E-39 |
| IL-10 | rs282258 | 2 | 224914800 | C | -0.0992 | 0.0162 | 1.00E-09 |
| IL-12p70 | rs145023524 | 6 | 43819046 | G | -0.279 | 0.0394 | 1.54E-12 |
| IL-12p70 | rs12199215 | 6 | 44026914 | C | -0.1278 | 0.0192 | 5.11E-11 |
| IL-12p70 | rs7754905 | 6 | 44150182 | G | 0.1029 | 0.019 | 4.28E-08 |
| IL-12p70 | rs2375980 | 9 | 2692622 | G | -0.0937 | 0.0159 | 4.55E-09 |
| IL-12p70 | rs7088799 | 10 | 65016174 | G | 0.0998 | 0.0161 | 6.29E-10 |
| IL-13 | rs9472168 | 6 | 43928985 | G | -0.4244 | 0.0248 | 1.08E-65 |
| IL-13 | rs75438658 | 6 | 44018321 | C | 0.343 | 0.0625 | 4.12E-08 |
| IL-16 | rs4253283 | 4 | 187165211 | C | -0.146 | 0.0262 | 1.75E-08 |
| IL-16 | rs1801020 | 5 | 176836532 | G | -0.1733 | 0.0272 | 4.53E-10 |
| IL-16 | rs4778636 | 15 | 81591639 | G | 0.7272 | 0.0633 | 1.11E-30 |
| IL-17 | rs1530455 | 3 | 122854899 | C | -0.108 | 0.0173 | 4.87E-10 |
| IL-18 | rs385076 | 2 | 32489851 | C | 0.2432 | 0.0248 | 1.66E-22 |
| IL-18 | rs116656892 | 5 | 68186028 | C | -0.5298 | 0.0925 | 1.05E-08 |
| IL-18 | rs115267715 | 5 | 68535015 | C | -0.4508 | 0.08 | 1.72E-08 |
| IL-18 | rs17229943 | 5 | 68682536 | C | 0.312 | 0.0463 | 1.62E-11 |
| IL-18 | rs71478720 | 11 | 112009605 | C | 0.2669 | 0.0276 | 3.71E-22 |
| IP-10 | rs113831257 | 4 | 76159521 | G | -0.3592 | 0.0644 | 2.53E-08 |
| IP-10 | rs9450351 | 6 | 86624320 | C | 0.2768 | 0.0489 | 1.48E-08 |
| MCP-1 | rs200154182 | 1 | 158872480 | TTTTC | -0.0965 | 0.0174 | 2.81E-08 |
| MCP-1 | rs2036297 | 3 | 46172903 | G | -0.119 | 0.016 | 1.09E-13 |
| MCP-1 | rs138591554 | 3 | 46289206 | T | 0.3171 | 0.0331 | 7.94E-22 |
| M-CSF | rs56367447 | 8 | 3871527 | C | 0.4967 | 0.0883 | 1.72E-08 |
| MIF | rs2330634 | 22 | 24250795 | G | -0.1556 | 0.0249 | 4.53E-10 |
| MIG | rs55876513 | 4 | 76883698 | G | -0.166 | 0.0255 | 8.23E-11 |
| MIP-1b | rs142242702 | 3 | 44164152 | T | 0.2454 | 0.0426 | 8.05E-09 |
| MIP-1b | rs62242542 | 3 | 45169491 | C | 0.4714 | 0.0425 | 1.05E-28 |
| MIP-1b | rs33802 | 3 | 45316089 | C | 0.1173 | 0.0165 | 1.20E-12 |
| MIP-1b | rs191600590 | 3 | 45610258 | T | -0.1502 | 0.0246 | 8.79E-10 |
| MIP-1b | rs79091774 | 3 | 45906878 | C | 0.4606 | 0.0751 | 8.83E-10 |
| MIP-1b | rs113010081 | 3 | 46457412 | C | 0.5954 | 0.0236 | 3.85E-140 |
| MIP-1b | rs12487651 | 3 | 46714708 | G | -0.1397 | 0.0193 | 3.81E-13 |
| MIP-1b | rs28827623 | 3 | 46830204 | G | -0.2752 | 0.0317 | 3.00E-18 |
| MIP-1b | rs77481436 | 3 | 47506230 | G | -0.1682 | 0.0259 | 7.97E-11 |
| MIP-1b | rs75485436 | 3 | 47935009 | G | -0.3426 | 0.0283 | 1.07E-33 |
| MIP-1b | rs75394422 | 3 | 49333079 | C | 0.3582 | 0.0408 | 1.83E-18 |
| MIP-1b | rs183139656 | 3 | 50399645 | C | -0.3095 | 0.0408 | 3.41E-14 |
| MIP-1b | rs78266901 | 3 | 51431525 | G | -0.2079 | 0.0336 | 5.17E-10 |
| MIP-1b | rs72829264 | 17 | 32832526 | G | 0.1601 | 0.0278 | 9.38E-09 |
| MIP-1b | rs4796047 | 17 | 33507219 | G | -0.1055 | 0.0156 | 1.38E-11 |
| MIP-1b | rs117620244 | 17 | 33648381 | C | 0.3528 | 0.0495 | 1.87E-12 |
| MIP-1b | rs62079535 | 17 | 33665329 | G | -0.238 | 0.039 | 1.05E-09 |
| MIP-1b | rs113699401 | 17 | 33668796 | G | -0.1474 | 0.0212 | 3.35E-12 |
| MIP-1b | rs71381463 | 17 | 33937266 | C | 0.1578 | 0.0257 | 6.80E-10 |
| MIP-1b | rs117715247 | 17 | 33978654 | G | 0.351 | 0.059 | 3.09E-09 |
| MIP-1b | rs76960253 | 17 | 34088656 | C | -0.5233 | 0.0585 | 5.45E-19 |
| MIP-1b | rs11651172 | 17 | 34270288 | G | 0.1366 | 0.0245 | 2.11E-08 |
| MIP-1b | rs6505501 | 17 | 34347238 | C | 0.1556 | 0.0191 | 3.71E-16 |
| MIP-1b | rs60516659 | 17 | 34403297 | G | -0.2691 | 0.0248 | 3.64E-27 |
| MIP-1b | rs113877493 | 17 | 34812273 | C | 0.6124 | 0.0218 | 1.62E-173 |
| MIP-1b | rs111942332 | 17 | 34818508 | G | 0.4727 | 0.0573 | 1.70E-16 |
| MIP-1b | rs76842834 | 17 | 34883848 | C | 0.4206 | 0.0472 | 7.33E-19 |
| MIP-1b | rs17693183 | 17 | 34964290 | G | 0.5795 | 0.0795 | 8.93E-13 |
| MIP-1b | rs9330240 | 17 | 34974689 | C | 0.4745 | 0.046 | 5.84E-25 |
| MIP-1b | rs10491120 | 17 | 34988444 | G | -0.3001 | 0.0318 | 5.15E-21 |
| MIP-1b | rs80007108 | 17 | 35012096 | C | -0.2259 | 0.031 | 2.73E-13 |
| MIP-1b | rs1867288 | 17 | 35021348 | G | -0.2079 | 0.0215 | 5.94E-22 |
| MIP-1b | rs72820246 | 17 | 35039992 | G | 0.1042 | 0.0168 | 5.40E-10 |
| MIP-1b | rs117453826 | 17 | 35132809 | G | 0.5774 | 0.0593 | 5.07E-22 |
| MIP-1b | rs4795162 | 17 | 35236530 | G | -0.1261 | 0.0158 | 1.14E-15 |
| MIP-1b | rs7213769 | 17 | 36115166 | G | -0.1008 | 0.017 | 3.14E-09 |
| MIP-1b | rs7221878 | 17 | 36191133 | C | 0.3045 | 0.0463 | 7.37E-11 |
| PDGF-bb | rs12990266 | 2 | 224306859 | G | -0.2363 | 0.0342 | 3.18E-12 |
| PDGF-bb | rs13412535 | 2 | 224874874 | G | -0.3352 | 0.0214 | 2.46E-55 |
| PDGF-bb | rs13024765 | 2 | 225180219 | C | 0.1014 | 0.0158 | 1.14E-10 |
| PDGF-bb | rs2324229 | 6 | 83918131 | C | -0.0894 | 0.0161 | 3.48E-08 |
| PDGF-bb | rs4965869 | 15 | 101990320 | C | -0.184 | 0.0181 | 5.66E-24 |
| PDGF-bb | rs9806745 | 15 | 102010311 | C | -0.1162 | 0.0163 | 1.10E-12 |
| PDGF-bb | rs28406863 | 15 | 102086276 | G | 0.2089 | 0.0382 | 4.78E-08 |
| RANTES | rs74472919 | 13 | 82200650 | C | -0.3313 | 0.0605 | 3.97E-08 |
| SCF | rs1557570 | 1 | 169507844 | G | -0.1186 | 0.017 | 2.74E-12 |
| SCF | rs4841899 | 9 | 137424412 | C | 0.1004 | 0.0178 | 1.78E-08 |
| SCGF-β | rs4656185 | 1 | 169476326 | G | -0.205 | 0.0256 | 1.16E-15 |
| SCGF-β | rs17876031 | 5 | 176831119 | G | 0.1514 | 0.0255 | 2.25E-09 |
| SCGF-β | rs73185877 | 12 | 103773440 | G | -0.5249 | 0.0711 | 1.18E-13 |
| SCGF-β | rs117716477 | 12 | 104240958 | C | -0.8384 | 0.0841 | 1.34E-23 |
| SCGF-β | rs181218758 | 12 | 104496721 | C | 0.3833 | 0.0586 | 5.58E-11 |
| SCGF-β | rs116924815 | 19 | 51230733 | C | -0.6079 | 0.0738 | 1.74E-16 |
| TNF-β | rs78296352 | 1 | 22821844 | G | -1.2215 | 0.1366 | 4.76E-21 |
| TNF-β | rs116196280 | 1 | 23047050 | G | -0.7179 | 0.1006 | 4.98E-13 |
| TRAIL | rs3136596 | 3 | 172228872 | G | -0.1147 | 0.0209 | 3.65E-08 |
| TRAIL | rs79287178 | 3 | 172294500 | G | 0.4317 | 0.0421 | 9.12E-25 |
| TRAIL | rs183815186 | 18 | 25336963 | T | 0.3564 | 0.0604 | 3.52E-09 |
| TRAIL | rs57396456 | 18 | 27945877 | C | 0.5626 | 0.0518 | 1.25E-27 |
| TRAIL | rs74778900 | 18 | 28086266 | C | -0.5906 | 0.0532 | 2.59E-28 |
| TRAIL | rs193112415 | 18 | 28835120 | C | 1.0421 | 0.0623 | 2.15E-62 |
| TRAIL | rs62093514 | 18 | 29230977 | C | -1.0618 | 0.0552 | 6.86E-82 |
| TRAIL | rs77451439 | 18 | 29399005 | G | 0.4322 | 0.0369 | 1.21E-31 |
| TRAIL | rs664216 | 18 | 29425152 | T | 0.1385 | 0.0231 | 2.02E-09 |
| TRAIL | rs9952273 | 18 | 29575063 | C | -0.864 | 0.0499 | 3.86E-69 |
| TRAIL | rs11081739 | 18 | 29583126 | G | -0.1411 | 0.0202 | 3.34E-12 |
| TRAIL | rs62093947 | 18 | 29660305 | C | 0.7596 | 0.046 | 3.31E-61 |
| TRAIL | rs679163 | 18 | 29822893 | G | -0.1066 | 0.0162 | 4.24E-11 |
| TRAIL | rs138987090 | 18 | 30366247 | G | 0.7497 | 0.0752 | 4.50E-23 |
| TRAIL | rs72965213 | 18 | 32028143 | C | -0.3636 | 0.0566 | 9.37E-11 |
| VEGF | rs9381249 | 6 | 43734798 | C | 0.2482 | 0.0397 | 3.09E-10 |
| VEGF | rs6920532 | 6 | 43793430 | C | -0.1803 | 0.0267 | 8.68E-12 |
| VEGF | rs67798973 | 6 | 43882777 | G | -0.1389 | 0.0175 | 1.29E-15 |
| VEGF | rs6921438 | 6 | 43925607 | G | 0.49 | 0.0175 | 2.09E-171 |
| VEGF | rs34467391 | 6 | 43940371 | AG | 0.1293 | 0.0171 | 3.40E-14 |
| VEGF | rs74675876 | 6 | 43963995 | C | 0.2822 | 0.0366 | 7.62E-15 |
| VEGF | rs4507572 | 6 | 44135095 | C | -0.1007 | 0.0171 | 3.34E-09 |
| VEGF | rs41282660 | 6 | 44197006 | G | 0.1613 | 0.0263 | 1.33E-09 |
| VEGF | rs34881325 | 9 | 2622134 | C | 0.1082 | 0.0189 | 1.04E-08 |
| VEGF | rs7030781 | 9 | 2686273 | T | -0.1368 | 0.0173 | 2.57E-15 |
| VEGF | rs10761739 | 10 | 65062008 | G | -0.1182 | 0.0174 | 1.06E-11 |

Abbreviations: β-NGF, beta nerve growth factor; Chr, chromosome; CTACK, cutaneous T-cell attracting (CCL27); GRO-a, growth regulated oncogene-α (CXCL1); HGF, hepatocyte growth factor; IBD, inflammatory bowel disease; IFN-γ, Interferon-gamma; IL-2rα, interleukin-2 receptor, alpha subunit; IL-12p70, interleukin- 12p70; IL-13, interleukin-13; IL-16, interleukin-16; IL-17, interleukin-17; IL-18, interleukin-18;IP-10, Interferon gamma-induced protein 10 (CXCL10); MCP-1, monocyte chemotactic protein-1 (CCL2); M-CSF, macrophage colony-stimulating factor; MIF, macrophage migration inhibitory factor; MIG, monokine induced by interferon-gamma; MIP-1b, macrophage inflammatory protein-1β; PDGF-bb, platelet derived growth factor BB; RANTES, regulated on activation, normal T Cell expressed and secreted (CCL5); SCF, stem cell factor; SCGF-β, stem cell growth factor beta; SDF-1α, stromal cell-derived factor-1 alpha; SE, standard error; SNP, single nucleotide polymorphism; TNF-β, tumor necrosis factor-beta; TRAIL, TNF-related apoptosis inducing ligand; UC, ulcerative colitis; VEGF, vascular endothelial growth factor.

**Supplementary Table 6** Effect estimates of the associations between circulating levels of 28 cytokines and risk of inflammatory bowel disease.

| Cytokines/ Growth factors | The results of IBDGC | | | |  | The results of UK biobank | | | |
| --- | --- | --- | --- | --- | --- | --- | --- | --- | --- |
|  | N.SNPs | OR | 95% CI | *P-*value |  | N.SNPs | OR | 95% CI | *P-*value |
| **β-NGF** |  |  |  |  |  |  |  |  |  |
| Inverse-variance weighted | 1 | 0.88 | 0.70-1.11 | 0.280 |  | 1 | 1.01 | 0.81-1.26 | 0.917 |
| Weighted median | / | / | / | / |  | / | / | / | / |
| MR-PRESSO test | / | / | / | / |  | / | / | / | / |
| MR-Egger | / | / | / | / |  | / | / | / | / |
| **CTACK** |  |  |  |  |  |  |  |  |  |
| Inverse-variance weighted | 4 | 0.97 | 0.89-1.06 | 0.500 |  | 3 | 0.89 | 0.82-0.98 | 0.014 |
| Weighted median | 4 | 0.94 | 0.85-1.04 | 0.227 |  | 3 | 0.88 | 0.80-0.97 | 0.014 |
| MR-PRESSO test | 4 | 0.97 | 0.90-1.05 | 0.542 |  | / | / | / | / |
| MR-Egger | 4 | / | / | 0.242* |  | 3 | / | / | 0.802* |
| **Eotaxin** |  |  |  |  |  |  |  |  |  |
| Inverse-variance weighted | 3 | 0.96 | 0.79-1.17 | 0.710 |  | 3 | 0.83 | 0.69-1.01 | 0.067 |
| Weighted median | 3 | 0.97 | 0.77-1.21 | 0.781 |  | 3 | 0.95 | 0.73-1.23 | 0.687 |
| MR-PRESSO test | / | / | / | / |  | / | / | / | / |
| MR-Egger | 3 | / | / | 0.937* |  | 3 | / | / | 0.953* |
| **GRO-a** |  |  |  |  |  |  |  |  |  |
| Inverse-variance weighted | 1 | 0.97 | 0.88-1.07 | 0.516 |  | 1 | 1.10 | 0.99-1.21 | 0.077 |
| Weighted median | / | / | / | / |  | / | / | / | / |
| MR-PRESSO test | / | / | / | / |  | / |  | / | / |
| MR-Egger | / | / | / | / |  | / | / | / | / |
| **HGF** |  |  |  |  |  |  |  |  |  |
| Inverse-variance weighted | 2 | 1.02 | 0.85-1.22 | 0.864 |  | 2 | 0.92 | 0.77-1.10 | 0.345 |
| Weighted median | / | / | / | / |  | / | / | / | / |
| MR-PRESSO test | / | / | / | / |  | / | / | / | / |
| MR-Egger | / | / | / | / |  | / | / | / | / |
| **IFN-γ** |  |  |  |  |  |  |  |  |  |
| Inverse-variance weighted | 1 | 1.17 | 0.63-2.14 | 0.620 |  | 1 | 0.89 | 0.60-1.33 | 0.572 |
| Weighted median | / | / | / | / |  | / | / | / | / |
| MR-PRESSO test | / | / | / | / |  | / | / | / | / |
| MR-Egger | / | / | / | / |  | / | / | / | / |
| **IL2ra** |  |  |  |  |  |  |  |  |  |
| Inverse-variance weighted | 1 | 1.02 | 0.94-1.11 | 0.588 |  | 1 | 1.10 | 1.00-1.21 | 0.040 |
| Weighted median | / | / | / | / |  | / | / | / | / |
| MR-PRESSO test | / | / | / | / |  | / | / | / | / |
| MR-Egger | / | / | / | / |  | / | / | / | / |
| **IL-5** |  |  |  |  |  |  |  |  |  |
| Inverse-variance weighted | 1 | 1.05 | 0.84-1.30 | 0.686 |  | 1 | 0.78 | 0.63-0.97 | 0.028 |
| Weighted median | / | / | / | / |  | / | / | / | / |
| MR-PRESSO test | / | / | / | / |  | / | / | / | / |
| MR-Egger | / | / | / | / |  | / | / | / | / |
| **IL-7** |  |  |  |  |  |  |  |  |  |
| Inverse-variance weighted | 1 | 1.02 | 0.92-1.13 | 0.697 |  | 1 | 0.88 | 0.79-0.98 | 0.018 |
| Weighted median | / | / | / | / |  | / | / | / | / |
| MR-PRESSO test | / | / | / | / |  | / | / | / | / |
| MR-Egger | / | / | / | / |  | / | / | / | / |
| **IL-10** |  |  |  |  |  |  |  |  |  |
| Inverse-variance weighted | 1 | 1.13 | 0.80-1.58 | 0.489 |  | 1 | 1.00 | 0.71-1.41 | 0.989 |
| Weighted median | / | / | / | / |  | / | / | / | / |
| MR-PRESSO test | / | / | / | / |  | / | / | / | / |
| MR-Egger | / | / | / | / |  | / | / | / | / |
| **IL-12p70** |  |  |  |  |  |  |  |  |  |
| Inverse-variance weighted | 5 | 1.04 | 0.88-1.23 | 0.650 |  | 4 | 1.02 | 0.86-1.21 | 0.788 |
| Weighted median | 5 | 1.09 | 0.88-1.35 | 0.423 |  | 4 | 1.01 | 0.82-1.25 | 0.926 |
| MR-PRESSO test | 5 | 1.04 | 0.90-1.21 | 0.628 |  | 4 | 1.02 | 0.80-1.31 | 0.865 |
| MR-Egger | 5 | / | / | 0.351* |  | 4 | / | / | 0.813* |
| **IL-13** |  |  |  |  |  |  |  |  |  |
| Inverse-variance weighted | 2 | 1.01 | 0.93-1.10 | 0.772 |  | 1 | 1.35 | 0.85-2.13 | 0.200 |
| Weighted median | / | / | / | / |  | / | / | / | / |
| MR-PRESSO test | / | / | / | / |  | / | / | / | / |
| MR-Egger | / | / | / | / |  | / | / | / | / |
| **IL-16** |  |  |  |  |  |  |  |  |  |
| Inverse-variance weighted | 3 | 1.01 | 0.94-1.09 | 0.720 |  | 3 | 1.00 | 0.93-1.07 | 0.943 |
| Weighted median | 3 | 1.01 | 0.93-1.09 | 0.803 |  | 3 | 0.99 | 0.92-1.07 | 0.827 |
| MR-PRESSO test | / | / | / | / |  | / | / | / | / |
| MR-Egger | 3 | / | / | 0.554* |  | 3 | / | / | 0.759* |
| **IL-17** |  |  |  |  |  |  |  |  |  |
| Inverse-variance weighted | 1 | 1.52 | 1.10-2.08 | 0.010 |  | 1 | 1.08 | 0.78-1.49 | 0.643 |
| Weighted median | / | / | / | / |  | / | / | / | / |
| MR-PRESSO test | / | / | / | / |  | / | / | / | / |
| MR-Egger | / | / | / | / |  | / | / | / | / |
| **IL-18** |  |  |  |  |  |  |  |  |  |
| Inverse-variance weighted | 5 | 1.13 | 0.97-1.32 | 0.104 |  | 4 | 1.08 | 0.98-1.19 | 0.106 |
| Weighted median | 5 | 1.22 | 1.09-1.36 | 0.000 |  | 4 | 1.09 | 0.97-1.24 | 0.154 |
| MR-PRESSO test | 4 | 1.13 | 0.97-1.32 | 0.179 |  | 4 | 1.08 | 0.95-1.24 | 0.337 |
| MR-Egger | 5 | / | / | 0.001* |  | 4 | / | / | 0.018* |
| **IP-10** |  |  |  |  |  |  |  |  |  |
| Inverse-variance weighted | 2 | 0.96 | 0.80-1.14 | 0.614 |  | 2 | 1.05 | 0.88-1.25 | 0.561 |
| Weighted median | / | / | / | / |  | / | / | / | / |
| MR-PRESSO test | / | / | / | / |  | / | / | / | / |
| MR-Egger | / | / | / | / |  | / | / | / | / |
| **MCP-1** |  |  |  |  |  |  |  |  |  |
| Inverse-variance weighted | 2 | 0.94 | 0.80-1.10 | 0.420 |  | 1 | 1.07 | 0.79-1.43 | 0.672 |
| Weighted median | / | / | / | / |  | / | / | / | / |
| MR-PRESSO test | / | / | / | / |  | / | / | / | / |
| MR-Egger | / | / | / | / |  | / | / | / | / |
| **M-CSF** |  |  |  |  |  |  |  |  |  |
| Inverse-variance weighted | 1 | 1.08 | 0.91-1.28 | 0.398 |  | 1 | 1.00 | 0.83-1.19 | 0.962 |
| Weighted median | / | / | / | / |  | / | / | / | / |
| MR-PRESSO test | / | / | / | / |  | / | / | / | / |
| MR-Egger | / | / | / | / |  | / | / | / | / |
| **MIF** |  |  |  |  |  |  |  |  |  |
| Inverse-variance weighted | 1 | 0.90 | 0.72-1.11 | 0.326 |  | 1 | 0.97 | 0.78-1.21 | 0.786 |
| Weighted median | / | / | / | / |  | / | / | / | / |
| MR-PRESSO test | / | / | / | / |  | / | / | / | / |
| MR-Egger | / | / | / | / |  | / | / | / | / |
| **MIG** |  |  |  |  |  |  |  |  |  |
| Inverse-variance weighted | 1 | 1.58 | 1.24-2.00 | 0.000 |  | 1 | 1.13 | 0.89-1.43 | 0.316 |
| Weighted median | / | / | / | / |  | / | / | / | / |
| MR-PRESSO test | / | / | / | / |  | / | / | / | / |
| MR-Egger | / | / | / | / |  | / | / | / | / |
| **MIP-1β** |  |  |  |  |  |  |  |  |  |
| Inverse-variance weighted | 37 | 1.05 | 0.99-1.10 | 0.083 |  | 31 | 1.02 | 0.98-1.06 | 0.325 |
| Weighted median | 37 | 1.03 | 0.96-1.11 | 0.362 |  | 31 | 1.04 | 0.97-1.11 | 0.251 |
| MR-PRESSO test | 36 | 1.05 | 0.99-1.10 | 0.091 |  | 31 | 1.02 | 0.98-1.06 | 0.340 |
| MR-Egger | 37 | / | / | 0.162* |  | 31 | / | / | 0.889* |
| **PDGF-bb** |  |  |  |  |  |  |  |  |  |
| Inverse-variance weighted | 7 | 0.98 | 0.90-1.08 | 0.731 |  | 6 | 1.00 | 0.91-1.09 | 0.916 |
| Weighted median | 7 | 0.97 | 0.86-1.09 | 0.569 |  | 6 | 1.02 | 0.93-1.13 | 0.651 |
| MR-PRESSO test | 7 | 0.98 | 0.90-1.08 | 0.735 |  | 6 | 1.00 | 0.92-1.08 | 0.915 |
| MR-Egger | 7 | / | / | 0.696* |  | 6 | / | / | 0.417* |
| **RANTES** |  |  |  |  |  |  |  |  |  |
| Inverse-variance weighted | 1 | 1.01 | 0.76-1.36 | 0.923 |  | 1 | 0.86 | 0.62-1.19 | 0.368 |
| Weighted median | / | / | / | / |  | / | / | / | / |
| MR-PRESSO test | / | / | / | / |  | / | / | / | / |
| MR-Egger | / | / | / | / |  | / | / | / | / |
| **SCF** |  |  |  |  |  |  |  |  |  |
| Inverse-variance weighted | 2 | 0.98 | 0.78-1.22 | 0.837 |  | 2 | 1.01 | 0.80-1.27 | 0.929 |
| Weighted median | / | / | / | / |  | / | / | / | / |
| MR-PRESSO test | / | / | / | / |  | / | / | / | / |
| MR-Egger | / | / | / | / |  | / | / | / | / |
| **SCGF-β** |  |  |  |  |  |  |  |  |  |
| Inverse-variance weighted | 6 | 1.03 | 0.95-1.12 | 0.508 |  | 5 | 1.02 | 0.89-1.16 | 0.824 |
| Weighted median | 6 | 1.05 | 0.95-1.15 | 0.357 |  | 5 | 1.04 | 0.93-1.16 | 0.483 |
| MR-PRESSO test | 6 | 1.03 | 0.97-1.09 | 0.398 |  | 4 | 1.02 | 0.89-1.16 | 0.835 |
| MR-Egger | 6 | / | / | 0.685* |  | 5 | / | / | 0.448* |
| **TNF-β** |  |  |  |  |  |  |  |  |  |
| Inverse-variance weighted | 2 | 0.99 | 0.91-1.07 | 0.750 |  | 2 | 0.98 | 0.92-1.05 | 0.645 |
| Weighted median | / | / | / | / |  | / | / | / | / |
| Simple median | / | / | / | / |  | / | / | / | / |
| MR-Egger | / | / | / | / |  | / | / | / | / |
| **TRAIL** |  |  |  |  |  |  |  |  |  |
| Inverse-variance weighted | 15 | 1.03 | 0.98-1.09 | 0.234 |  | 12 | 0.95 | 0.90-0.99 | 0.027 |
| Weighted median | 15 | 1.05 | 0.98-1.12 | 0.171 |  | 12 | 0.94 | 0.88-1.00 | 0.060 |
| MR-PRESSO test | 15 | 1.03 | 0.97-1.10 | 0.332 |  | 12 | 0.95 | 0.90-1.00 | 0.065 |
| MR-Egger | 15 | / | / | 0.969* |  | 12 | / | / | 0.555* |
| **VEGF** |  |  |  |  |  |  |  |  |  |
| Inverse-variance weighted | 10 | 1.06 | 0.97-1.16 | 0.198 |  | 7 | 0.96 | 0.86-1.08 | 0.527 |
| Weighted median | 10 | 1.03 | 0.97-1.10 | 0.357 |  | 7 | 0.93 | 0.88-1.00 | 0.036 |
| MR-PRESSO test | 9 | 1.06 | 0.97-1.16 | 0.230 |  | 6 | 0.96 | 0.86-1.08 | 0.550 |
| MR-Egger | 10 | / | / | 0.547* |  | 7 | / | / | 0.311* |

Abbreviations: β-NGF, beta nerve growth factor; CI, confidence interval; CTACK, cutaneous T-cell attracting (CCL27); GRO-a, growth regulated oncogene-α (CXCL1); HGF, hepatocyte growth factor; IBD, inflammatory bowel disease; IIBDGC, international inflammatory bowel disease genetics consortium; IFN-γ, interferon-gamma; IL-2rα, interleukin-2 receptor, alpha subunit; IL-5, interleukin-5; IL-7, interleukin-7; IL-8, interleukin-8; IL-10, interleukin-10; IL-12p70, interleukin-12p70; IL-13, interleukin-13; IL-16, interleukin-16; IL-17, interleukin-17; IL-18, interleukin-18; IP-10, interferon gamma-induced protein 10 (CXCL10); MCP-1, monocyte chemotactic protein-1; M-CSF, macrophage colony-stimulating factor; MIF, macrophage migration inhibitory factor; MIG, monokine induced by interferon-gamma; MIP-1b, macrophage inflammatory protein-1β; MR, Mendelian randomization; MR-PRESSO,MR pleiotropy residual sum and outlier; No., number of; OR, odds ratio; PDGF-bb, platelet derived growth factor BB; RANTES, regulated on activation normal T Cell expressed and secreted (CCL5); SCF, stem cell factor; SCGF-β, stem cell growth factor beta; SNP, single nucleotide polymorphism; TNF-β, tumor necrosis factor-beta; TRAIL, TNF-related apoptosis inducing ligand; VEGF, vascular endothelial growth factor. **P*-value of the intercept from MR-Egger regression analysis.

**Supplementary Table 7** Effect estimates of the associations between circulating levels of 28 cytokines and risk of Crohn's disease.

| Cytokines/ Growth factors | No. SNPs | OR | 95% CI | *P-*value |
| --- | --- | --- | --- | --- |
| **β-NGF** |  |  |  |  |
| Inverse-variance weighted | 1 | 0.71 | 0.52-0.98 | 0.039 |
| Weighted median | / | / | / | / |
| MR-PRESSO test | / | / | / | / |
| MR-Egger | / | / | / | / |
| **CTACK** |  |  |  |  |
| Inverse-variance weighted | 4 | 0.99 | 0.89-1.11 | 0.885 |
| Weighted median | 4 | 0.95 | 0.82-1.09 | 0.465 |
| MR-PRESSO test | 4 | 0.99 | 0.86-1.14 | 0.915 |
| MR-Egger | 4 | / | / | 0.091* |
| **Eotaxin** |  |  |  |  |
| Inverse-variance weighted | 3 | 1.02 | 0.78-1.34 | 0.876 |
| Weighted median | 3 | 0.96 | 0.66-1.38 | 0.819 |
| MR-PRESSO test | / | / | / | / |
| MR-Egger | 3 | / | / | 0.691* |
| **GRO-a** |  |  |  |  |
| Inverse-variance weighted | 1 | 0.97 | 0.85-1.11 | 0.647 |
| Weighted median | / | / | / | / |
| MR-PRESSO test | / | / | / | / |
| MR-Egger | / | / | / | / |
| **HGF** |  |  |  |  |
| Inverse-variance weighted | 2 | 1.12 | 0.88-1.44 | 0.351 |
| Weighted median | / | / | / | / |
| MR-PRESSO test | / | / | / | / |
| MR-Egger | / | / | / | / |
| **IFN-γ** |  |  |  |  |
| Inverse-variance weighted | 1 | 1.59 | 0.63-4.03 | 0.331 |
| Weighted median | / | / | / | / |
| MR-PRESSO test | / | / | / | / |
| MR-Egger | / | / | / | / |
| **IL2ra** |  |  |  |  |
| Inverse-variance weighted | 1 | 1.04 | 0.92-1.16 | 0.542 |
| Weighted median | / | / | / | / |
| MR-PRESSO test | / | / | / | / |
| MR-Egger | / | / | / | / |
| **IL-5** |  |  |  |  |
| Inverse-variance weighted | 1 | 1.20 | 0.90-1.62 | 0.216 |
| Weighted median | / | / | / | / |
| MR-PRESSO test | / | / | / | / |
| MR-Egger | / | / | / | / |
| **IL-7** |  |  |  |  |
| Inverse-variance weighted | 1 | 1.10 | 0.95-1.26 | 0.201 |
| Weighted median | / | / | / | / |
| MR-PRESSO test | / | / | / | / |
| MR-Egger | / | / | / | / |
| **IL-10** |  |  |  |  |
| Inverse-variance weighted | 1 | 0.95 | 0.60-1.51 | 0.821 |
| Weighted median | / | / | / | / |
| MR-PRESSO test | / | / | / | / |
| MR-Egger | / | / | / | / |
| **IL-12p70** |  |  |  |  |
| Inverse-variance weighted | 5 | 1.03 | 0.82-1.30 | 0.797 |
| Weighted median | 5 | 0.94 | 0.71-1.26 | 0.694 |
| MR-PRESSO test | 5 | 1.03 | 0.85-1.25 | 0.774 |
| MR-Egger | 5 | / | / | 0.964* |
| **IL-13** |  |  |  |  |
| Inverse-variance weighted | 2 | 1.07 | 0.97-1.20 | 0.188 |
| Weighted median | / | / | / | / |
| MR-PRESSO test | / | / | / | / |
| MR-Egger | / | / | / | / |
| **IL-16** |  |  |  |  |
| Inverse-variance weighted | 3 | 1.08 | 0.98-1.20 | 0.136 |
| Weighted median | 3 | 1.08 | 0.97-1.20 | 0.167 |
| MR-PRESSO test | / | / | / | / |
| MR-Egger | 3 | / | / | 0.892* |
| **IL-17** |  |  |  |  |
| Inverse-variance weighted | 1 | 1.70 | 1.10-2.63 | 0.016 |
| Weighted median | / | / | / | / |
| MR-PRESSO test | / | / | / | / |
| MR-Egger | / | / | / | / |
| **IL-18** |  |  |  |  |
| Inverse-variance weighted | 5 | 1.09 | 0.97-1.23 | 0.151 |
| Weighted median | 5 | 1.13 | 0.98-1.31 | 0.086 |
| MR-PRESSO test | 5 | 1.09 | 0.96-1.24 | 0.247 |
| MR-Egger | 5 | / | / | 0.110* |
| **IP-10** |  |  |  |  |
| Inverse-variance weighted | 2 | 0.90 | 0.71-1.14 | 0.370 |
| Weighted median | / | / | / | / |
| MR-PRESSO test | / | / | / | / |
| MR-Egger | / | / | / | / |
| **MCP-1** |  |  |  |  |
| Inverse-variance weighted | 2 | 1.06 | 0.86-1.31 | 0.599 |
| Weighted median | / | / | / | / |
| MR-PRESSO test | / | / | / | / |
| MR-Egger | / | / | / | / |
| **M-CSF** |  |  |  |  |
| Inverse-variance weighted | 1 | 1.04 | 0.83-1.31 | 0.713 |
| Weighted median | / | / | / | / |
| MR-PRESSO test | / | / | / | / |
| MR-Egger | / | / | / | / |
| **MIF** |  |  |  |  |
| Inverse-variance weighted | 1 | 0.96 | 0.72-1.29 | 0.808 |
| Weighted median | / | / | / | / |
| MR-PRESSO test | / | / | / | / |
| MR-Egger | / | / | / | / |
| **MIG** |  |  |  |  |
| Inverse-variance weighted | 1 | 1.71 | 1.24-2.36 | 0.001 |
| Weighted median | / | / | / | / |
| MR-PRESSO test | / | / | / | / |
| MR-Egger | / | / | / | / |
| **MIP-1β** |  |  |  |  |
| Inverse-variance weighted | 37 | 1.01 | 0.96-1.07 | 0.633 |
| Weighted median | 37 | 0.98 | 0.89-1.07 | 0.598 |
| MR-PRESSO test | 37 | 1.01 | 0.95-1.08 | 0.688 |
| MR-Egger | 37 | / | / | 0.043* |
| **PDGF-bb** |  |  |  |  |
| Inverse-variance weighted | 7 | 1.01 | 0.89-1.15 | 0.896 |
| Weighted median | 7 | 1.00 | 0.86-1.17 | 0.981 |
| MR-PRESSO test | / | / | / | / |
| MR-Egger | 7 | / | / | 0.453* |
| **RANTES** |  |  |  |  |
| Inverse-variance weighted | 1 | 1.15 | 0.78-1.72 | 0.476 |
| Weighted median | / | / | / | / |
| MR-PRESSO test | / | / | / | / |
| MR-Egger | / | / | / | / |
| **SCF** |  |  |  |  |
| Inverse-variance weighted | 2 | 0.94 | 0.69-1.27 | 0.678 |
| Weighted median | / | / | / | / |
| MR-PRESSO test | / | / | / | / |
| MR-Egger | / | / | / | / |
| **SCGF-β** |  |  |  |  |
| Inverse-variance weighted | 6 | 0.99 | 0.89-1.11 | 0.926 |
| Weighted median | 6 | 1.00 | 0.88-1.13 | 0.964 |
| MR-PRESSO test | 6 | 0.99 | 0.94-1.05 | 0.865 |
| MR-Egger | 6 | / | / | 0.911* |
| **TNF-β** |  |  |  |  |
| Inverse-variance weighted | 2 | 0.90 | 0.80-1.01 | 0.070 |
| Weighted median | / | / | / | / |
| MR-PRESSO test | / | / | / | / |
| MR-Egger | / | / | / | / |
| **TRAIL** |  |  |  |  |
| Inverse-variance weighted | 15 | 1.03 | 0.96-1.10 | 0.441 |
| Weighted median | 15 | 1.04 | 0.94-1.14 | 0.455 |
| MR-PRESSO test | 15 | 1.03 | 0.96-1.10 | 0.432 |
| MR-Egger | 15 | / | / | 0.755* |
| **VEGF** |  |  |  |  |
| Inverse-variance weighted | 10 | 1.07 | 0.99-1.16 | 0.078 |
| Weighted median | 10 | 1.07 | 0.98-1.17 | 0.153 |
| MR-PRESSO test | 10 | 1.07 | 0.98-1.18 | 0.181 |
| MR-Egger | 10 | / | / | 0.611* |

Abbreviations: β-NGF, beta nerve growth factor; CI, confidence interval; CTACK, cutaneous T-cell attracting (CCL27); GRO-a, growth regulated oncogene-α (CXCL1); HGF, hepatocyte growth factor; IFN-γ, interferon-gamma; IL-2rα, interleukin-2 receptor, alpha subunit; IL-5, interleukin-5; IL-7, interleukin-7; IL-8, interleukin-8; IL-10, interleukin-10; IL-12p70, interleukin-12p70; IL-13, interleukin-13; IL-16, interleukin-16; IL-17, interleukin-17; IL-18, interleukin-18; IP-10, interferon gamma-induced protein 10 (CXCL10); MCP-1, monocyte chemotactic protein-1; M-CSF, macrophage colony-stimulating factor; MIF, macrophage migration inhibitory factor; MIG, monokine induced by interferon-gamma; MIP-1b, macrophage inflammatory protein-1β; MR, Mendelian randomization; MR-PRESSO,MR pleiotropy residual sum and outlier; No., number of; OR, odds ratio; PDGF-bb, platelet derived growth factor BB; RANTES, regulated on activation normal T Cell expressed and secreted (CCL5); SCF, stem cell factor; SCGF-β, stem cell growth factor beta; SNP, single nucleotide polymorphism; TNF-β, tumor necrosis factor-beta; TRAIL, TNF-related apoptosis inducing ligand; VEGF, vascular endothelial growth factor. **P*-value of the intercept from MR-Egger regression analysis.

**Supplementary Table 8** Effect estimates of the associations between circulating levels of 28 cytokines and risk of ulcerative colitis.

| Cytokines/ Growth factors | No. SNPs | OR | 95% CI | *P-*value |
| --- | --- | --- | --- | --- |
| **β-NGF** |  |  |  |  |
| Inverse-variance weighted | 1 | 0.97 | 0.73-1.29 | 0.819 |
| Weighted median | / | / | / | / |
| MR-PRESSO test | / | / | / | / |
| MR-Egger | / | / | / | / |
| **CTACK** |  |  |  |  |
| Inverse-variance weighted | 4 | 0.95 | 0.86-1.05 | 0.340 |
| Weighted median | 4 | 0.91 | 0.80-1.04 | 1.155 |
| MR-PRESSO test | 4 | 0.95 | 0.87-1.04 | 0.368 |
| MR-Egger | 4 | / | / | 0.235* |
| **Eotaxin** |  |  |  |  |
| Inverse-variance weighted | 3 | 0.91 | 0.64-1.29 | 0.603 |
| Weighted median | 3 | 1.00 | 0,71-1.40 | 0.997 |
| MR-PRESSO test | / | / | / | / |
| MR-Egger | 3 | / | / | 0.770* |
| **GRO-a** |  |  |  |  |
| Inverse-variance weighted | 1 | 0.99 | 0.87-1.12 | 0.845 |
| Weighted median | / | / | / | / |
| MR-PRESSO test | / | / | / | / |
| MR-Egger | / | / | / | / |
| **HGF** |  |  |  |  |
| Inverse-variance weighted | 2 | 0.91 | 0.73-1.15 | 0.449 |
| Weighted median | / | / | / | / |
| MR-PRESSO test | / | / | / | / |
| MR-Egger | / | / | / | / |
| **IFN-γ** |  |  |  |  |
| Inverse-variance weighted | 1 | 1.01 | 0.49-2.07 | 0.977 |
| Weighted median | / | / | / | / |
| MR-PRESSO test | / | / | / | / |
| MR-Egger | / | / | / | / |
| **IL2ra** |  |  |  |  |
| Inverse-variance weighted | 1 | 1.03 | 0.92-1.15 | 0.607 |
| Weighted median | / | / | / | / |
| MR-PRESSO test | / | / | / | / |
| MR-Egger | / | / | / | / |
| **IL-5** |  |  |  |  |
| Inverse-variance weighted | 1 | 0.96 | 0.73-1.27 | 0.800 |
| Weighted median | / | / | / | / |
| MR-PRESSO test | / | / | / | / |
| MR-Egger | / | / | / | / |
| **IL-7** |  |  |  |  |
| Inverse-variance weighted | 1 | 0.98 | 0.86-1.12 | 0.815 |
| Weighted median | / | / | / | / |
| MR-PRESSO test | / | / | / | / |
| MR-Egger | / | / | / | / |
| **IL-10** |  |  |  |  |
| Inverse-variance weighted | 1 | 1.22 | 0.80-1.88 | 0.357 |
| Weighted median | / | / | / | / |
| MR-PRESSO test | / | / | / | / |
| MR-Egger | / | / | / | / |
| **IL-12p70** |  |  |  |  |
| Inverse-variance weighted | 5 | 1.04 | 0.84-1.29 | 0.693 |
| Weighted median | 5 | 1.20 | 0.91-1.58 | 0.196 |
| MR-PRESSO test | 5 | 1.04 | 0.86-1.27 | 0.692 |
| MR-Egger | 5 | / | / | 0.121* |
| **IL-13** |  |  |  |  |
| Inverse-variance weighted | 2 | 0.98 | 0.89-1.08 | 0.696 |
| Weighted median | / | / | / | / |
| MR-PRESSO test | / | / | / | / |
| MR-Egger | / | / | / | / |
| **IL-16** |  |  |  |  |
| Inverse-variance weighted | 3 | 1.00 | 0.91-1.09 | 0.953 |
| Weighted median | 3 | 1.00 | 0.90-1.10 | 0.920 |
| MR-PRESSO test | / | / | / | / |
| MR-Egger | 3 | / | / | 0.947* |
| **IL-17** |  |  |  |  |
| Inverse-variance weighted | 1 | 1.37 | 0.92-2.05 | 0.119 |
| Weighted median | / | / | / | / |
| MR-PRESSO test | / | / | / | / |
| MR-Egger | / | / | / | / |
| **IL-18** |  |  |  |  |
| Inverse-variance weighted | 5 | 1.14 | 0.93-1.39 | 0.206 |
| Weighted median | 5 | 1.24 | 1.09-1.43 | 0.002 |
| MR-PRESSO test | 4 | 1.14 | 0.93-1.39 | 0.275 |
| MR-Egger | 5 | / | / | 0.001* |
| **IP-10** |  |  |  |  |
| Inverse-variance weighted | 2 | 1.01 | 0.58-1.75 | 0.985 |
| Weighted median | / | / | / | / |
| MR-PRESSO test | / | / | / | / |
| MR-Egger | / | / | / | / |
| **MCP-1** |  |  |  |  |
| Inverse-variance weighted | 2 | 0.86 | 0.71-1.06 | 0.155 |
| Weighted median | / | / | / | / |
| MR-PRESSO test | / | / | / | / |
| MR-Egger | / | / | / | / |
| **M-CSF** |  |  |  |  |
| Inverse-variance weighted | 1 | 1.12 | 0.91-1.39 | 0.292 |
| Weighted median | / | / | / | / |
| MR-PRESSO test | / | / | / | / |
| MR-Egger | / | / | / | / |
| **MIF** |  |  |  |  |
| Inverse-variance weighted | 1 | 0.88 | 0.67-1.15 | 0.349 |
| Weighted median | / | / | / | / |
| MR-PRESSO test | / | / | / | / |
| MR-Egger | / | / | / | / |
| **MIG** |  |  |  |  |
| Inverse-variance weighted | 1 | 1.44 | 1.07-1.94 | 0.016 |
| Weighted median | / | / | / | / |
| MR-PRESSO test | / | / | / | / |
| MR-Egger | / | / | / | / |
| **MIP-1β** |  |  |  |  |
| Inverse-variance weighted | 37 | 1.08 | 1.01-1.15 | 0.019 |
| Weighted median | 37 | 1.03 | 0.95-1.13 | 0.463 |
| MR-PRESSO test | 36 | 1.08 | 1.01-1.15 | 0.024 |
| MR-Egger | 37 | / | / | 0.626* |
| **PDGF-bb** |  |  |  |  |
| Inverse-variance weighted | 7 | 0.95 | 0.84-1.06 | 0.349 |
| Weighted median | 7 | 0.95 | 0.82-1.11 | 0.530 |
| MR-PRESSO test | 7 | 0.95 | 0.85-1.05 | 0.351 |
| MR-Egger | 7 | / | / | 0.974* |
| **RANTES** |  |  |  |  |
| Inverse-variance weighted | 1 | 0.89 | 0.61-1.29 | 0.533 |
| Weighted median | / | / | / | / |
| MR-PRESSO test | / | / | / | / |
| MR-Egger | / | / | / | / |
| **SCF** |  |  |  |  |
| Inverse-variance weighted | 2 | 0.95 | 0.71-1.26 | 0.703 |
| Weighted median | / | / | / | / |
| MR-PRESSO test | / | / | / | / |
| MR-Egger | / | / | / | / |
| **SCGF-β** |  |  |  |  |
| Inverse-variance weighted | 6 | 1.03 | 0.93-1.14 | 0.595 |
| Weighted median | 6 | 1.03 | 0.91-1.17 | 0.642 |
| MR-PRESSO test | 6 | 1.03 | 0.94-1.13 | 0.573 |
| MR-Egger | 6 | / | / | 0.971* |
| **TNF-β** |  |  |  |  |
| Inverse-variance weighted | 2 | 1.05 | 0.96-1.15 | 0.313 |
| Weighted median | / | / | / | / |
| MR-PRESSO test | / | / | / | / |
| MR-Egger | / | / | / | / |
| **TRAIL** |  |  |  |  |
| Inverse-variance weighted | 15 | 1.04 | 0.98-1.11 | 0.203 |
| Weighted median | 15 | 1.07 | 0.98-1.17 | 0.109 |
| MR-PRESSO test | 15 | 1.04 | 0.97-1.12 | 0.298 |
| MR-Egger | 15 | / | / | 0.739* |
| **VEGF** |  |  |  |  |
| Inverse-variance weighted | 10 | 1.05 | 0.94-1.17 | 0.410 |
| Weighted median | 10 | 1.01 | 0.93-1.10 | 0.857 |
| MR-PRESSO test | 9 | 1.05 | 0.94-1.17 | 0.432 |
| MR-Egger | 10 | / | / | 0.688* |

Abbreviations: β-NGF, beta nerve growth factor; CI, confidence interval; CTACK, cutaneous T-cell attracting (CCL27); GRO-a, growth regulated oncogene-α (CXCL1); HGF, hepatocyte growth factor; IFN-γ, interferon-gamma; IL-2rα, interleukin-2 receptor, alpha subunit; IL-5, interleukin-5; IL-7, interleukin-7; IL-8, interleukin-8; IL-10, interleukin-10; IL-12p70, interleukin-12p70; IL-13, interleukin-13; IL-16, interleukin-16; IL-17, interleukin-17; IL-18, interleukin-18; IP-10, interferon gamma-induced protein 10 (CXCL10); MCP-1, monocyte chemotactic protein-1; M-CSF, macrophage colony-stimulating factor; MIF, macrophage migration inhibitory factor; MIG, monokine induced by interferon-gamma; MIP-1b, macrophage inflammatory protein-1β; MR, Mendelian randomization; MR-PRESSO,MR pleiotropy residual sum and outlier; No., number of; OR, odds ratio; PDGF-bb, platelet derived growth factor BB; RANTES, regulated on activation normal T Cell expressed and secreted (CCL5); SCF, stem cell factor; SCGF-β, stem cell growth factor beta; SNP, single nucleotide polymorphism; TNF-β, tumor necrosis factor-beta; TRAIL, TNF-related apoptosis inducing ligand; VEGF, vascular endothelial growth factor. **P*-value of the intercept from MR-Egger regression analysis.

**Supplementary Table 9** Effect estimates of the associations between inflammatory bowel disease and circulating levels of IL-17 and MIG.

| Cytokines/ Growth factors | NO. SNPs | OR | 95% CI | *P-*value |
| --- | --- | --- | --- | --- |
| IL-17 |  |  |  |  |
| Inverse-variance weighted | 38 | 0.98 | 0.94-1.02 | 0.374 |
| Weighted median | 38 | 0.98 | 0.92-1.03 | 0.423 |
| MR-PRESSO test | 38 | 0.98 | 0.94-1.02 | 0.376 |
| MR-Egger | 38 | / | / | 0.033* |
| MIG |  |  |  |  |
| Inverse-variance weighted | 38 | 1.01 | 0.94-1.08 | 0.864 |
| Weighted median | 38 | 0.97 | 0.89-1.06 | 0.531 |
| MR-PRESSO test | 38 | 1.01 | 0.94-1.08 | 0.865 |
| MR-Egger | 38 | / | / | 0.444* |

Abbreviations: CI, confidence interval; IL-17, interleukin-17; MIG, monokine induced by interferon-gamma; MR-PRESSO test, MR Pleiotropy RESidual Sum and Outlier test; No., number of; OR, odds ratio; SNP, single nucleotide polymorphism; SNP, single nucleotide polymorphism. **P*-value of the intercept from MR-Egger regression analysis.

**Supplementary Table 10** Effect estimates of the associations of Crohn's disease with risk of circulating levels of β-NGF, , IL-17 and MIG.

| Cytokines/ Growth factors | No. SNPs | OR | 95% CI | *P-*value |
| --- | --- | --- | --- | --- |
| β-NGF |  |  |  |  |
| Inverse-variance weighted | 30 | 1.06 | 1.00-1.13 | 0.063 |
| Weighted median | 30 | 1.06 | 0.98-1.15 | 0.137 |
| MR-PRESSO test | 30 | 1.06 | 1.00-1.13 | 0.074 |
| MR-Egger | 30 | / | / | 0.177* |
| IL-17 |  |  |  |  |
| Inverse-variance weighted | 31 | 1.00 | 0.97-1.04 | 0.820 |
| Weighted median | 31 | 1.00 | 0.95-1.06 | 0.918 |
| MR-PRESSO test | 31 | 1.00 | 0.97-1.04 | 0.800 |
| MR-Egger | 31 | / | / | 0.118* |
| MIG |  |  |  |  |
| Inverse-variance weighted | 30 | 1.03 | 0.97-1.10 | 0.343 |
| Weighted median | 30 | 1.05 | 0.97-1.13 | 0.263 |
| MR-PRESSO test | 30 | 1.03 | 0.97-1.10 | 0.351 |
| MR-Egger | 30 | / | / | 0.125* |

Abbreviations: β-NGF, beta nerve growth factor; CI, confidence interval; IL-17, interleukin-17; MIG, monokine induced by interferon-gamma; MR-PRESSO test, MR Pleiotropy RESidual Sum and Outlier test; No., number of; OR, odds ratio; SNP, single nucleotide polymorphism; SNP, single nucleotide polymorphism. **P*-value of the intercept from MR-Egger regression analysis.

**Supplementary Table 11** Effect estimates of the associations of ulcerative colitis with risk of circulating levels of MIG and MIP-1β.

| Cytokines/ Growth factors | No. SNPs | OR | 95% CI | *P-*value |
| --- | --- | --- | --- | --- |
| MIG |  |  |  |  |
| Inverse-variance weighted | 28 | 0.98 | 0.93-1.03 | 0.386 |
| Weighted median | 28 | 0.93 | 0.86-1.00 | 0.066 |
| MR-PRESSO test | 28 | 0.98 | 0.93-1.03 | 0.424 |
| MR-Egger | 28 | / | / | 0.557* |
| MIP-1β |  |  |  |  |
| Inverse-variance weighted | 28 | 0.96 | 0.92-1.00 | 0.062 |
| Weighted median | 28 | 0.92 | 0.87-0.97 | 0.004 |
| MR-PRESSO test | 28 | 0.96 | 0.92-1.00 | 0.072 |
| MR-Egger | 28 | / | / | 0.148* |

Abbreviations: CI, confidence interval; MIG, monokine induced by interferon-gamma; MIP-1β,macrophage inflammatory protein-1β; MR-PRESSO test, MR Pleiotropy RESidual Sum and Outlier test; No., number of; OR, odds ratio; SNP, single nucleotide polymorphism; SNP, single nucleotide polymorphism. **P*-value of the intercept from MR-Egger regression analysis.


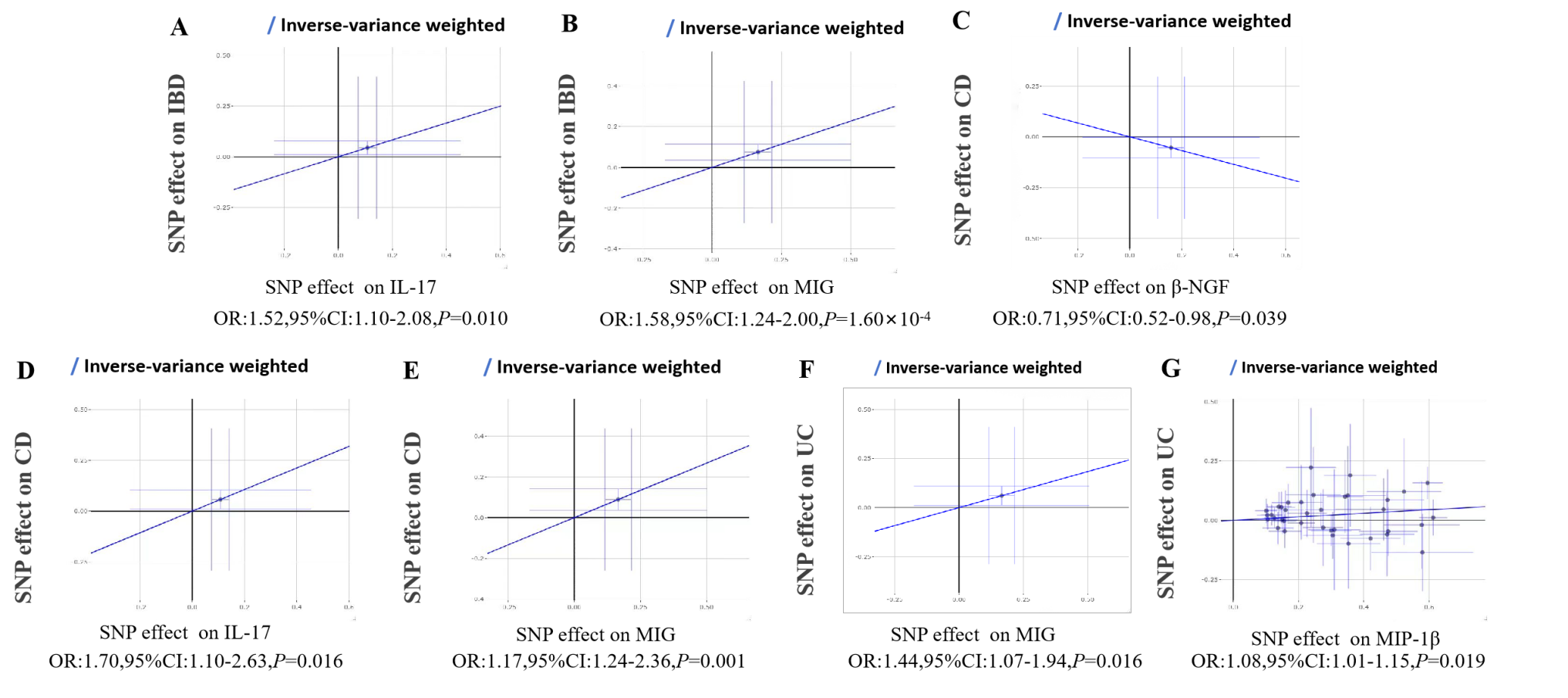


**Supplementary Figure 1.** **Scatter plot of the associations of genetic variants with circulating cytokines and the risk of inflammatory bowel disease, Crohn's disease and ulcerative colitis.**

Abbreviations: β-NGF, beta nerve growth factor; CD, Crohn's disease; CI, confidence interval; IBD, inflammatory bowel disease; IL-17, interleukin-17; MIG, monokine induced by interferon-gamma; MIP-1b, macrophage inflammatory protein-1β; OR, odds ratio; SNP, single nucleotide polymorphism; UC, ulcerative colitis.
